# Three-Dimensional Covalent Organic Framework with **scu-c** Topology for Drug Delivery

**DOI:** 10.1021/acsami.2c15152

**Published:** 2022-10-17

**Authors:** Saikat Das, Taishu Sekine, Haruna Mabuchi, Tsukasa Irie, Jin Sakai, Yu Zhao, Qianrong Fang, Yuichi Negishi

**Affiliations:** † Department of Applied Chemistry, Faculty of Science, Tokyo University of Science, Kagurazaka, Shinjuku-ku, Tokyo 162-8601, Japan; ‡ Zhejiang Engineering Laboratory for Green Syntheses and Applications of Fluorine-Containing Specialty Chemicals, Institute of Advanced Fluorine-Containing Materials, Zhejiang Normal University, Jinhua 321004, P. R. China; § State Key Laboratory of Inorganic Synthesis and Preparative Chemistry, Jilin University, Changchun 130012, P. R. China

**Keywords:** covalent organic framework, three-dimensional, topology, two-fold interpenetrated, drug delivery

## Abstract

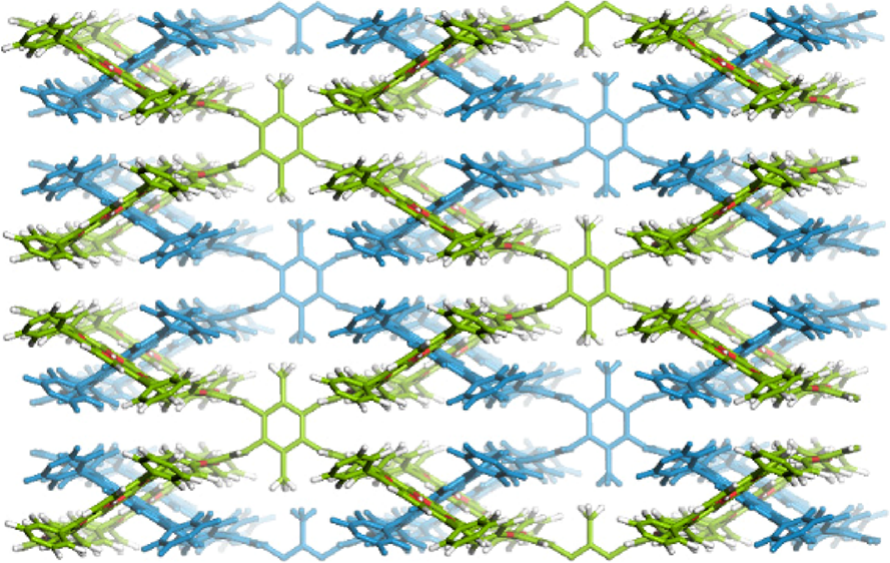

Three-dimensional (3D) covalent organic frameworks (COFs) exemplify a new generation of crystalline extended solids with intriguing structures and unprecedented porosity. Notwithstanding substantial scope, the reticular synthesis of 3D COFs from pre-designed building units leading to new network topologies yet remains a demanding task owing to the shortage of 3D building units and inadequate reversibility of the linkages between the building units. In this work, by linking a tetragonal prism (8-connected) node with a square planar (4-connected) node, we report the first 3D COF with **scu-c** topology. The new COF, namely, TUS-84, features a two-fold interpenetrated structure with well-defined porosity and a Brunauer–Emmett–Teller surface area of 679 m^2^ g^–1^. In drug delivery applications, TUS-84 shows efficient drug loading and sustained release profile.

## Introduction

An emerging class of porous organic materials developed from linking molecular building blocks with strong covalent bonds into crystalline, extended two-dimensional (2D) and three-dimensional (3D) structures called covalent organic frameworks (COFs)^1−14^ have recently aroused great interest in catalysis,^15,16^ sensing,^17,18^ separation,^19^ semiconduction,^20^ proton conduction,^21^ biomedicine,^22,23^ among others. COFs emerged in 2005^24^ as the second series of reticular materials, the first one being metal–organic frameworks (MOFs).^25,26^ “Reticular” means “anything that has the structure of a net”. By reticular synthesis, we refer to the extended structure regime that combines (i) molecular-level control over matter and (ii) robustness.^27−29^ A top-down reticular synthesis scheme starts with a desired net topology, followed by disassembling it into vertices and edges, finding secondary building units with the right connectivities and aligning them with the vertices, obtaining an augmented net by replacing the vertices of an *n*-connected net by a group of *n*-vertices, and finally linking the molecular building blocks by robust bonds into crystalline extended structures.^1,30−34^ Alternatively, the bottom-up scheme of reticular synthesis proceeds from pre-designed building units, leading to unprecedented network topologies.^35^ COFs feature one of the highest open-pore scaffolds. The COF scaffold is built out of organic units, and it imparts tunable chemical environments for encapsulating a wide array of guest molecules.

Based on the extension of their covalent connectivity, COFs can be categorized into 2D and 3D COFs.^36^ With covalent connectivity extending only in 2D, 2D COFs crystallize as layered structures in which the layers are stacked through non-covalent interactions (π–π stack, van der Waals interactions, hydrogen bonds), giving rise to one-dimensional (1D) straight channels.^14,37^ On the other hand, with covalent connectivity extending along the entire 3D scaffold, 3D COFs often have the upper hand over 2D COFs, attributed to their interconnected channels and readily accessible active sites.^38^

Topological consideration is crucial to 3D extended structures, considering that it largely dictates their pore architecture, active site formation, and mass transport behavior.^38^ Albeit highly sought after, discovery of new 3D COF topologies yet remains a herculean task because the highly connected 3D organic building blocks are hard to come by and it is very difficult to solve the crystal structures. Thus far, the type of 3D topologies of COFs is limited to about 20.^38^ The tetratopic (*T*_d_)-based 3D COF nets are **bor**,^39^**ctn**,^39^**dia**,^40^**pts**,^41^**rra**,^42^**lon**,^43^ and **ljh**.^44^ Fang et al. and He and et al. prepared several hexatopic (*D*_3h_)-based 3D COFs, for example, **stp**,^45^**acs**,^46,47^**ceq**,^47,48^ and **hea**.^49,50^ Different from triangular prismatic (*D*_3h_) nodes, triangular antiprismatic (*D*_3d_) nodes were utilized by Mateo-Alonso and co-workers to construct **pcu** topology 3D COFs.^51^ Other unprecedented 3D COF topologies reported are **ffc**,^52^**srs**,^53^**fjh**,^54^**tbo**,^55^ and **nbo**.^56^ Recently, octatopic nodes have been used to prepare **pcb**(57) and **bcu**(35) topology 3D COFs.

**Scheme 1:**
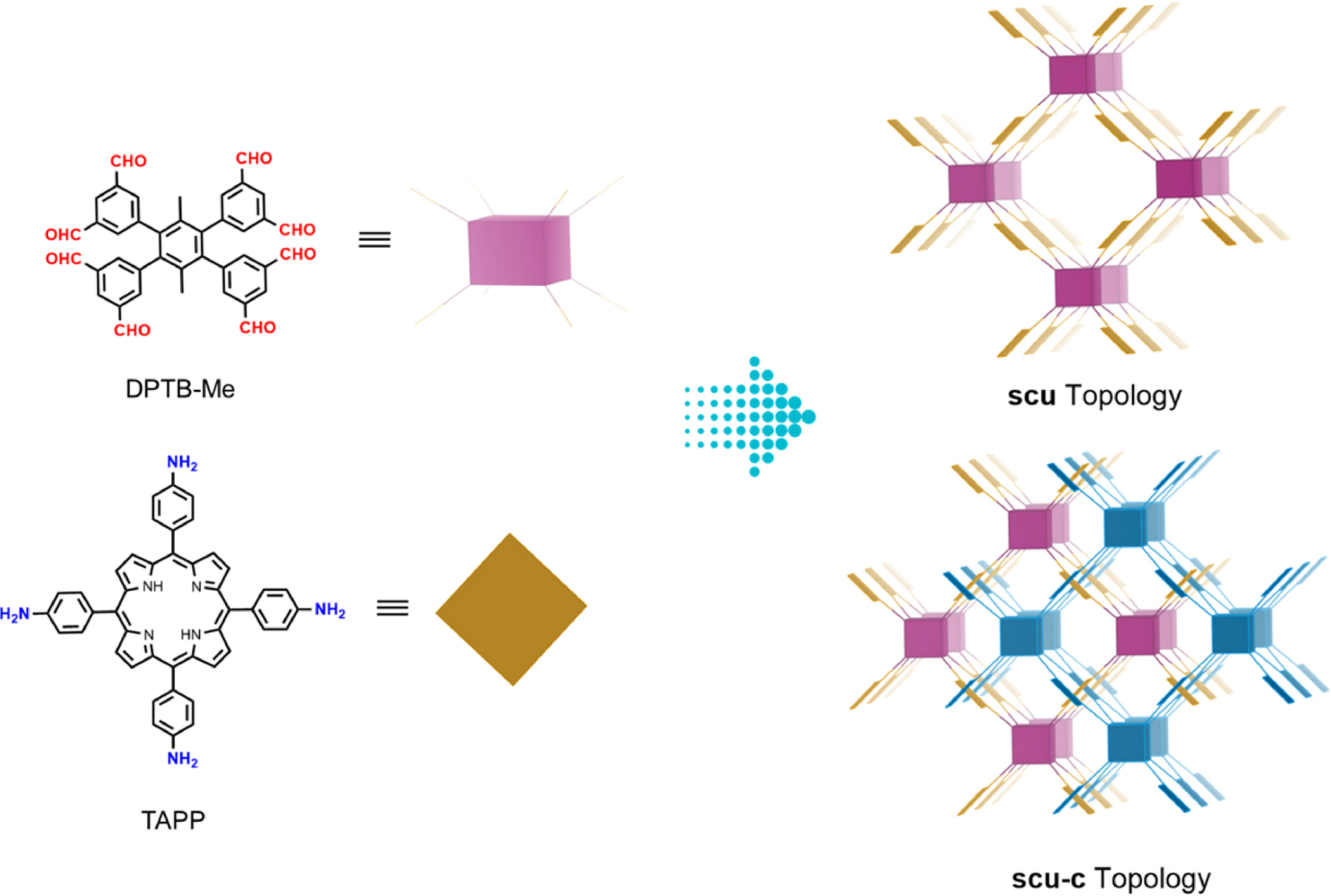
Strategy for Constructing 3D COF with the **scu** Topology^a^ The condensation reaction of a *D*_2h_-symmetric linker, DPTB-Me, and a *C*_4_-symmetric linker, TAPP, yielding 3D COF with a **scu-c** net, which belongs to **scu** topology.

Herein, we report the first^58^ 3D COF with **scu** topology, namely, TUS-84, formed through the combination of a tetragonal prism (8-connected) node with a square planar (4-connected) node. After this article was available online in open access preprint archive,^58^ a 3D COF with non-interpenetrated **scu** topology, NKCOF-25-H, constructed from the same strategy has been published.^59^ Differing from NKCOF-25-H with non-interpenetrated **scu** topology, TUS-84 exhibits a two-fold interpenetrated **scu** net, denoted as **scu-c** (c for catenated), and different unit cell parameters from NKCOF-25-H. Of note, the simulated non-interpenetrated **scu** net X-ray diffraction (XRD) pattern did not accord with our experimental powder XRD (PXRD) pattern, while the two-fold interpenetrated **scu** net XRD showed great alignment with our observed PXRD pattern. This case is similar to the non-interpenetrated **stp** COF (JUC-564)^45^ reported by Fang and co-workers and the 2-fold interpenetrated **stp** COF (Trip-COF 2)^60^ reported by Zhao and co-workers, both of which were synthesized using the same building blocks. Structural elucidation of TUS-84 was carried out thoroughly through different characterization techniques. Interestingly, TUS-84 shows efficient drug loading and extended release profile in simulated physiological media. Scheme 1 depicts the strategic approach to construct 3D COFs with a **scu-c** net via the [8 + 4] imine condensation reaction of a *D*_2h_-symmetric linker, 4′,5′-bis(3,5-diformylphenyl)-3′,6′-dimethyl-[1,1′:2′,1″-terphenyl]-3,3, ″5,5″-tetracarbaldehyde (DPTB-Me), and a *C*_4_-symmetric linker, 5,10,15,20-tetrakis(4-aminophenyl)porphyrin (TAPP).

## Results and Discussion

**Figure 1:**
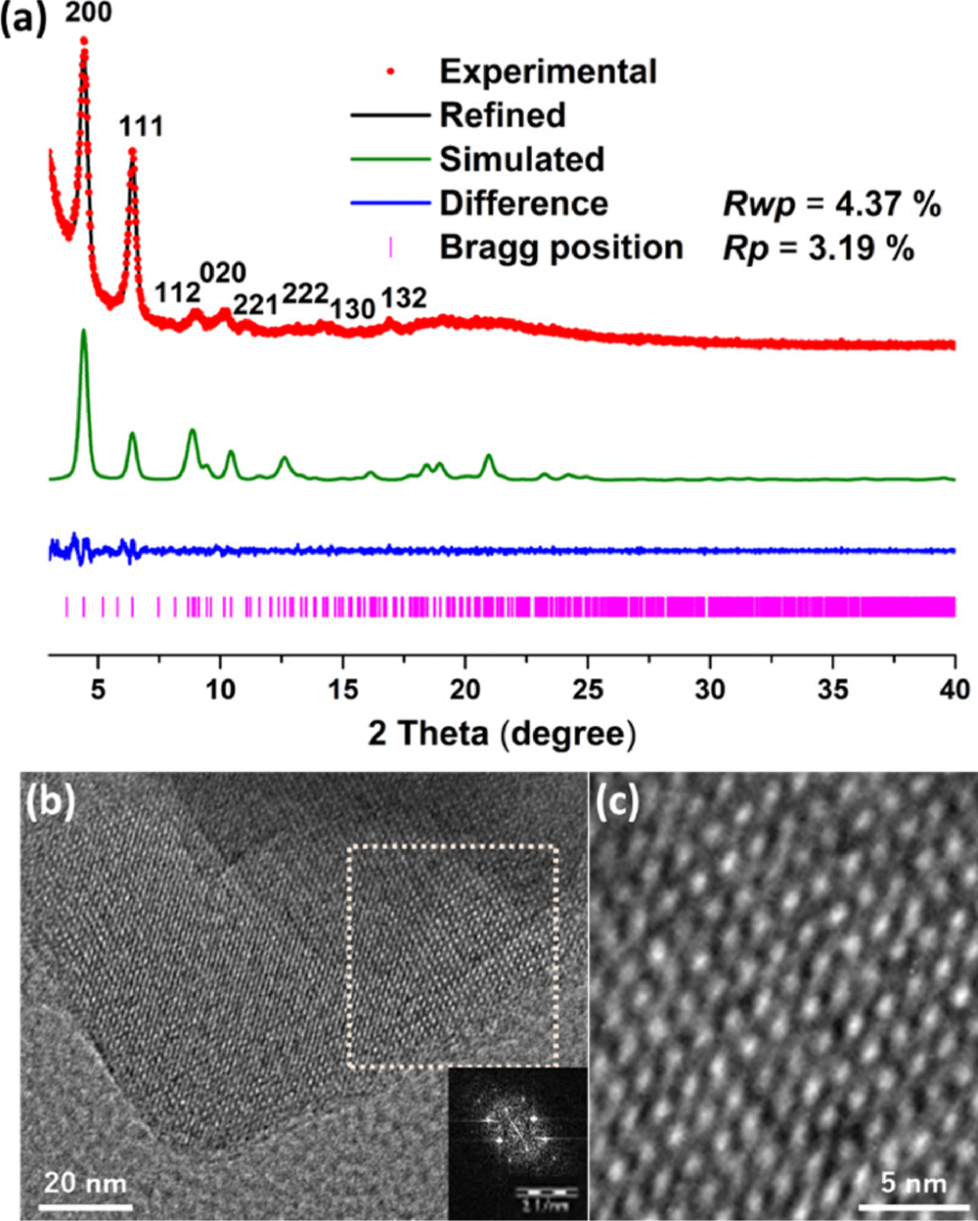
(a) PXRD patterns of TUS-84: experimental pattern (red dots), Pawley refined (black curve), simulated (green curve) pattern from the **scu-c** modeled structure, and the difference plot (blue curve) between the experimental and refined patterns. The Bragg positions are denoted by magenta ticks. (b, c) HRTEM images of TUS-84. The inset in (b) shows the fast Fourier transform (FFT) pattern acquired from the area enclosed by the white box.

**Figure 2:**
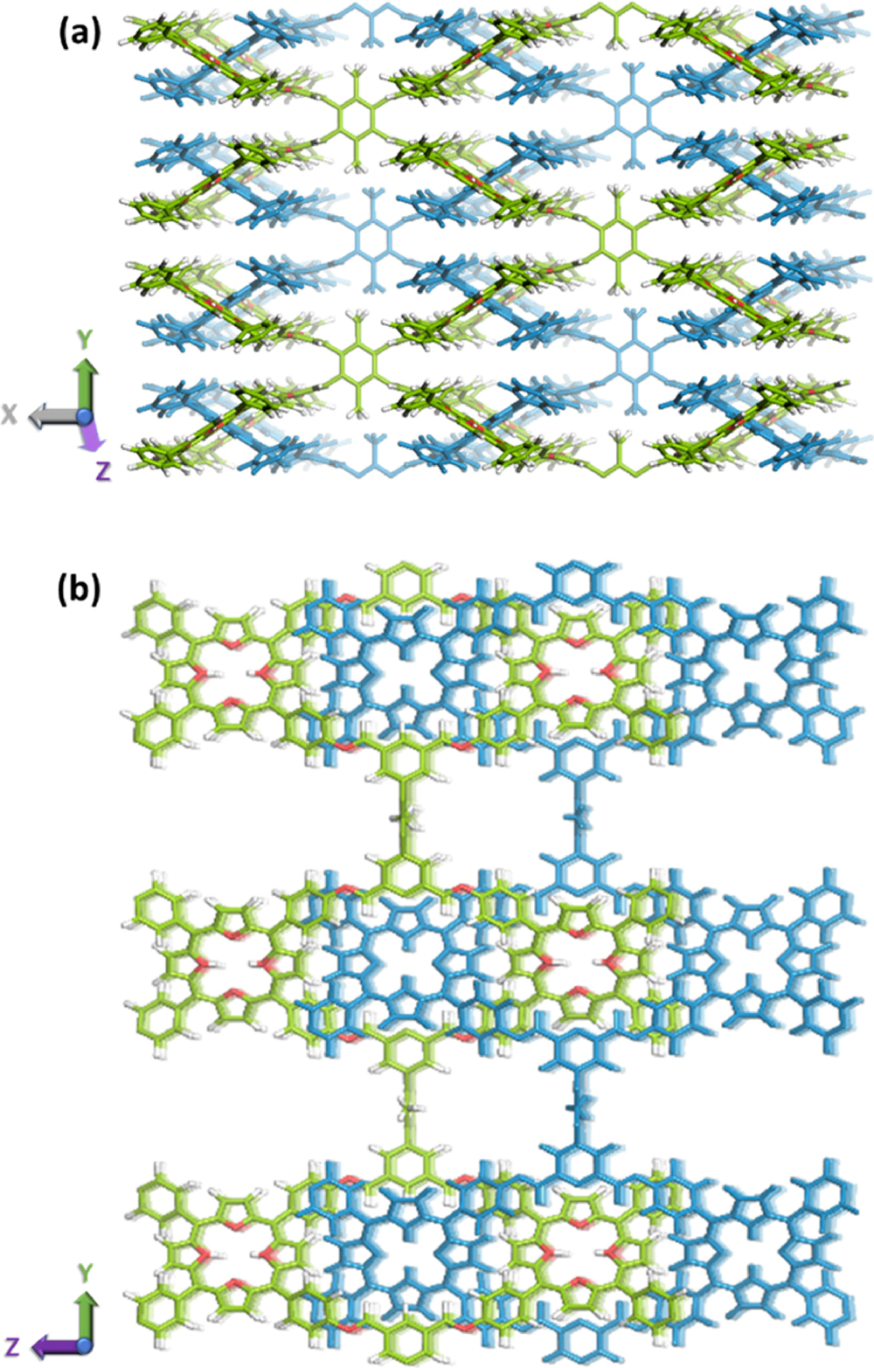
(a, b) Extended structures of TUS-84.

TUS-84 was synthesized by the solvothermal reaction of DPTB-Me (19.0 mg, 0.03 mmol) and TAPP (40.48 mg, 0.06 mmol) in a 5:5:2 (v/v/v) mixture of mesitylene, 1,4-dioxane, and 6 M aqueous acetic acid under 120 °C for 3 days. The acid-catalyzed Schiff-base condensation reaction yielded the COF as a dark purple crystalline solid at a yield of 76%. The solid-state ^13^C cross-polarization magic angle spinning (CP/MAS) NMR and Fourier-transform infrared (FT-IR) spectroscopies provided definitive evidence for atomic-level connectivity of the imine linkage in TUS-84. The ^13^C CP/MAS NMR spectrum displayed a characteristic peak at 158 ppm for the imine carbon of TUS-84 (Figure S1). In the FT-IR spectrum of TUS-84, the C=N vibration peak at 1625 cm^–1^ was observed. Significant attenuation of the N–H (3433, 3465 cm^–1^ for TAPP) and C=O (1703 cm^–1^ for DPTB-Me) stretching vibration bands in the FT-IR spectrum of TUS-84 implies a high degree of polymerization for the imine COF (Figure S2). Isometric microcrystals of TUS-84 were observed from scanning electron micrographs (Figure S3). High-resolution transmission electron microscopy (HRTEM) imaging (Figures 1b,c and S4,5) showed the ordered structure of TUS-84, comprising rhombus pores viewed along the *z*-direction in the simulated structure (Figure 2a). Thermogravimetric analysis (TGA) curve indicates high thermal stability for TUS-84 retaining 95% of its weight up to 500 °C (Figure S6). Chemical stability of the COF was substantiated from its preservation of crystallinity and imine linkage after treatment with organic solvents, water, and aqueous HCl and NaOH solutions, as can be seen from the PXRD profiles (Figure S7) and FT-IR spectra (Figure S8).

The crystal structure of TUS-84 was unraveled by PXRD analysis combined with structural modeling and simulation (Figure 1a). Geometry optimization (energy minimization) was performed in the *Materials Studio* 7.0^61^ Forcite program that afforded the unit cell parameters of TUS-84 with a **scu-c** net and *Pm* space group as *a* = 39.9205 Å, *b* = 18.7162 Å, *c* = 23.6564 Å, α = β = γ = 90°. The simulated PXRD pattern (Figure 1a, green curve) showed great alignment with the observed diffraction pattern (Figure 1a, red dots). Sharp Bragg peaks observed at 4.38 and 6.44° correspond to the (200) and (111) facets, respectively, and relatively weak peaks at 9.10, 10.23, 11.15, 12.83, 14.38, and 16.19° correspond to the (112), (020), (221), (222) (130), and (132) facets, respectively (Figure 1a). Pawley refinement was applied against the experimental PXRD data using Reflex that resulted in a space group of *Pm* with unit cell parameters *a* = 39.9179 Å, *b* = 18.7054 Å, *c* = 23.6772 Å, α = β = γ = 90° and good agreement factors *R*_p_ = 4.37%, *R*_wp_ = 3.19%. The Pawley refined PXRD pattern (black curve, Figure 1a) shows good consistency with the experimental PXRD pattern (red dots), as indicated by the minor difference plot (blue curve). Furthermore, we also explored alternative topologies for TUS-84, including the non-interpenetrated **scu** net (Figure S16, Table S2) and **csq** topology (Figure S17, Table S3). However, the simulated PXRD patterns did not accord with the experimental PXRD pattern. All things considered, we propose the **scu-c** net for TUS-84.

**Figure 3:**
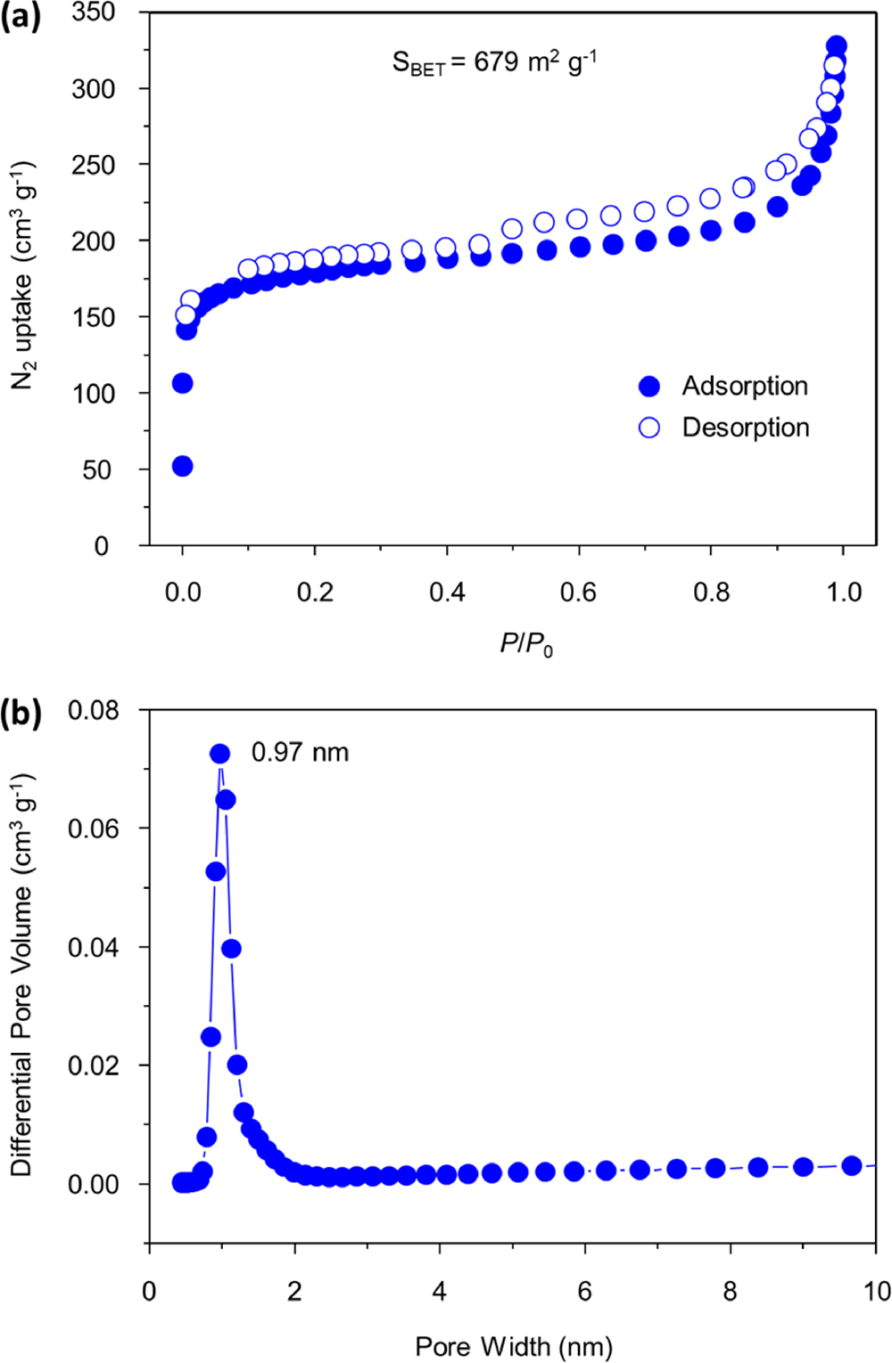
(a) Nitrogen sorption isotherms and (b) pore size distribution profile of TUS-84.

The permanent porosity of TUS-84 was ascertained by N_2_ sorption measurements on activated COF sample at 77 K. As can be observed in Figure 3a, TUS-84 displayed a reversible type-I isotherm with a sharp uptake at low pressure (*P*/*P*_0_ < 0.1), indicative of its microporous character. The BET specific surface area of TUS-84 was evaluated as 679 m^2^ g^–1^ (Figure S9). Applying the nonlocal density functional theory (NLDFT) method, the pore volume of TUS-84 was derived as 0.7613 cm^3^ g^–1^, and its pore size distribution was calculated as 0.97 nm (Figure 3b), consistent with the pore size predicted from the simulated structure (1.05 nm). We also evaluated the H_2_, CO_2_, and CH_4_ gas adsorption capacities of TUS-84 to reinforce its prospects in carbon capture and clean energy applications. As illustrated in Figure S10, the H_2_ uptake capacities at 77 and 87 K under 1 bar are 131 and 88 cm^3^ g^–1^, respectively. The isosteric enthalpy of adsorption (*Q*_st_) of H_2_ was calculated to be 6.8 kJ mol^–1^ (Figure S11). TUS-84 shows a CO_2_ uptake capacity of 55 and 31 cm^3^ g^–1^ at 273 and 298 K, respectively, under 1 bar (Figure S12). The *Q*_st_ of CO_2_ adsorption was evaluated as 24.9 kJ mol^–1^ (Figure S13). The CH_4_ sorption isotherms shown in Figure S14 reveal an uptake capacity of 14 and 10 cm^3^ g^–1^ at 273 and 298 K under 1 bar, respectively, and the value of *Q*_st_ was obtained as 6.5 kJ mol^–1^ (Figure S15).

Intrigued by the 3D functional scaffold with permanent porosity and high chemical stability, we utilized TUS-84 in in vitro drug delivery studies. Ibuprofen is one of the most common nonsteroidal anti-inflammatory drugs (NSAIDs) used for the treatment of rheumatoid arthritis, osteoarthritis, mild–moderate pain, and primary dysmenorrhea.^62−64^ The selection of ibuprofen as the drug in this study is based on the following: (a) ibuprofen has a short half-life (1.8–2.0 h) that calls for extended release formulations^62^ and (b) pore dimensions of TUS-84 (1.05 nm) is benefitting for encapsulation of ibuprofen with molecular size of 0.5 × 1 nm^2^.^65,66^

**Figure 4:**
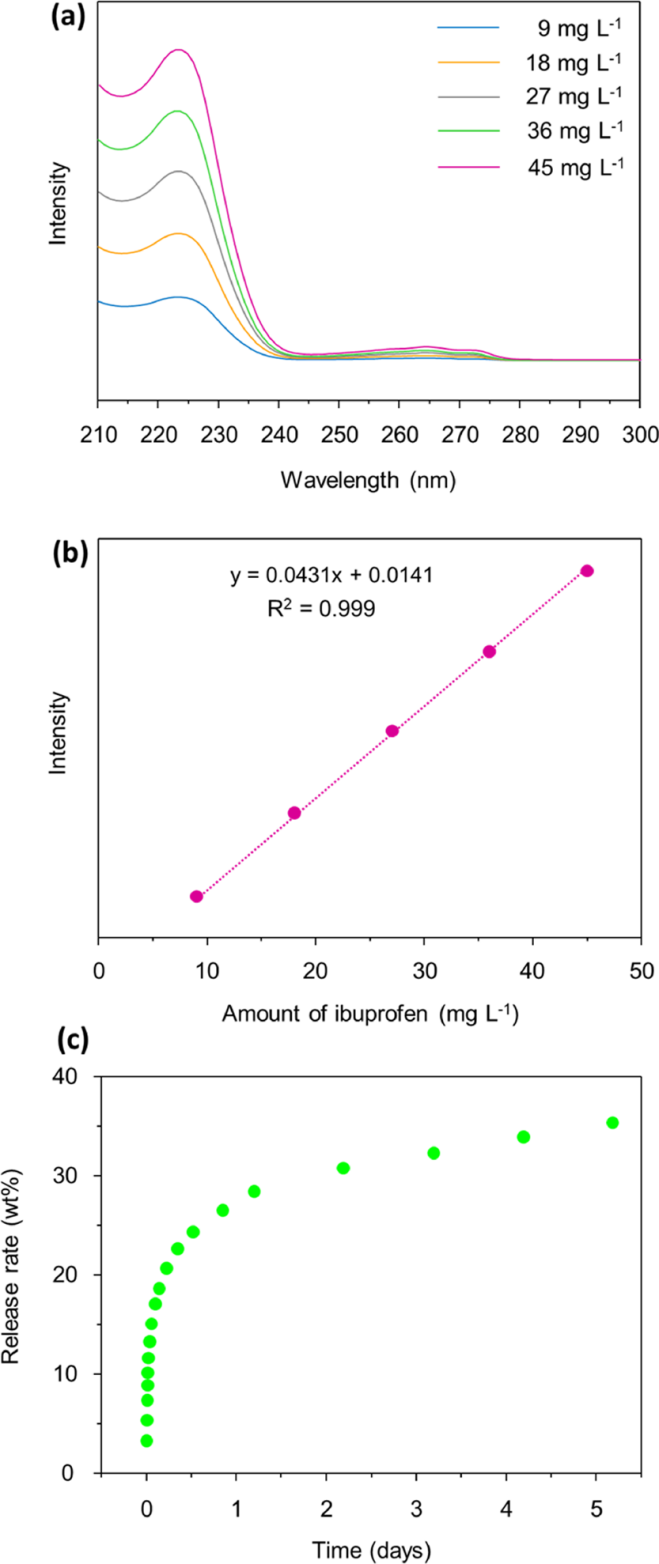
(a) UV–vis spectra of ibuprofen in simulated body fluid (pH 7.4, phosphate buffer solution) at different concentrations. (b) Calibration curve of ibuprofen. (c) Release profile of ibuprofen from ibuprofen-loaded TUS-84.

For drug loading, 50 mg of TUS-84 was suspended in 30 mL of 0.1 M hexane solution of ibuprofen under magnetic stirring for 4 h. The drug-loaded COF sample was isolated from suspension via vacuum filtration, washed with hexane, and subsequently dried at room temperature. 1.0 mL of the filtrate was collected and 50 times diluted to evaluate the loading amount of ibuprofen using a UV–vis spectrophotometer by measuring the absorbance at 261 nm of ibuprofen in hexane and supernatant (see Supporting Information for details). UV–vis absorption data showed 11.05 wt % loading of ibuprofen in TUS-84 (Figure S24). As can be seen from Figure S22, the value of drug loading amount is in good agreement with that obtained from TGA (11 wt %). PXRD analysis (Figure S18) of ibuprofen-loaded TUS-84 revealed that the COF crystalline structure was retained after drug loading. As evident from the scanning electron micrograph (Figure S19) of ibuprofen-loaded TUS-84, no significant morphological change in COF was observed. N_2_ sorption measurements of ibuprofen-loaded TUS-84 were carried out to ascertain the successful loading of ibuprofen inside the COF pores. The reduction in BET surface area from 679 to 462.7 m^2^ g^–1^ after ibuprofen loading was owing to the occupancy of COF pores by ibuprofen molecules. The drug release study was performed by placing 40 mg of the drug-loaded TUS-84 sample inside a semipermeable bag, followed by immersing in 10 mL of phosphate buffer solution (simulated body fluid, pH 7.4) at a constant temperature of 37 °C. The dissolution solvent was taken out at specified time intervals for evaluation of the ibuprofen concentration and replenished with 10 mL of fresh buffer solution. The ibuprofen concentration was determined UV–vis spectrophotometrically using a calibration curve (Figure 4a,b). TUS-84 showed an extended drug release performance of about 35% after 5 days (Figure 4c). In comparison with the ibuprofen release rates of 78% for Cage-COF-TT^67^ and 49% for PI-COF-5,^66^ respectively, after 12 h, a considerably slower release rate of 24% was observed for TUS-84 after 12 h. This long-acting ibuprofen formulation could deliver sustained concentrations of drug over a prolonged period of time, thereby reducing dosing frequency and ensuring more consistent control of long-lasting pains.^68,69^ For more detailed studies of TUS-84 on drug delivery, we also performed the loading and release of captopril. TGA trace of captopril-loaded TUS-84 revealed 16 wt % loading of captopril in TUS-84 (Figure S28). The majority of the captopril was released from TUS-84 after about 5 days, with total delivery reaching about 98% of the total captopril loading (Figure S29c).

## Conclusions

To conclude, a 3D COF with a novel **scu-c** topology was designed and synthesized, utilizing a *D*_2h_-symmetric linker, DPTB-Me, and a *C*_4_-symmetric linker, TAPP. The resultant TUS-84 COF displays an ordered microporous structure with high crystallinity and excellent stability. Furthermore, TUS-84 shows great promise as drug delivery vehicle owing to its efficient drug loading and controlled release behavior. This study may not only expand the library of 3D COF topologies but also facilitate the design of new 3D COF structures for biomedical applications.

## Supporting Information Available

Materials and characterization; synthetic procedures; solid-state ^13^C CP/MAS NMR spectroscopy; FT-IR spectroscopy; SEM and TEM images; TGA; chemical stability test; N_2_ adsorption; structure simulations and XRD analyses; drug delivery; and crystallographic information (PDF)
