# Supplementary material for: Three-Dimensional Covalent Organic Framework with scu-c Topology for Drug Delivery
Source: ACS Appl Mater Interfaces. 2022 Oct 17;14(42):48045–51. doi: 10.1021/acsami.2c15152 (PMC9614725; doi:10.1021/acsami.2c15152)
Supplement: Supplementary file 1 — am2c15152_si_001.pdf [file am2c15152_si_001.pdf]

[Supporting Information]

Three-Dimensional Covalent Organic Framework with scu-c Topology for Drug Delivery

Saikat Das,[†] Taishu Sekine,[†] Haruna Mabuchi,[†] Tsukasa Irie,[†] Jin Sakai,[†] Yu Zhao,[§] Qianrong Fang,[‡,*] and Yuichi Negishi[†,*]

[†]Department of Applied Chemistry, Faculty of Science, Tokyo University of Science, Kagurazaka, Shinjuku-ku, Tokyo 162-8601, Japan

[§]Zhejiang Engineering Laboratory for Green Syntheses and Applications of Fluorine-Containing Specialty Chemicals, Institute of Advanced Fluorine-Containing Materials, Zhejiang Normal University, Jinhua 321004, P. R. China

[‡]State Key Laboratory of Inorganic Synthesis and Preparative Chemistry, Jilin University, Changchun 130012, P. R. China

Corresponding Authors

[*]Qianrong Fang: qrfang@jlu.edu.cn

[*]Yuichi Negishi: negishi@rs.tus.ac.jp

Table Of Contents

Section 1. Materials and Characterization S3

Section 2. Synthetic procedures S5

Section 3. Solid-state ^{13}C CP/MAS Nuclear Magnetic Resonance (NMR) Spectroscopy S6

Section 4. Fourier-Transform Infrared (FT-IR) Spectroscopy S7

Section 5. Scanning electron microscopy (SEM) and Transmission electron microscopy (TEM) images S8

Section 6. Thermogravimetric analysis (TGA) S10

Section 7. Chemical stability test S11

Section 8. N_2 adsorption S13

Section 9. H_2, CO_2 and CH_4 adsorption S14

Section 10. Structure simulations and X-ray diffraction analyses S17

Section 11. Drug delivery S19

Section 12. Crystallographic information S28

Section 13. Liquid NMR spectra of building blocks S46

Section 14. References S50

1. Materials and Characterization

1.1. Materials. All reagents were of analytical grade as procured from commercial sources and used as received. The solvents were dried using conventional methods. All products were isolated and handled under nitrogen in glovebox or Schlenk line using standard techniques. Anhydrous mesitylene, 1,4-dioxane, and acetic acid were purchased from FUJIFILM Wako Pure Chemical Corporation. Anhydrous tetrahydrofuran, and dimethyl sulfoxide-d_6 were purchased from Kanto Chemical Co., Inc. Ibuprofen was purchased from Shanghai Macklin Biochemical Co., Ltd. 0.1 mol/L phosphate buffer solution (pH 7.4) was purchased from FUJIFILM Wako Pure Chemical Corporation.

1.2. Instruments.

Powder X-ray diffraction (PXRD) patterns were obtained on a Rigaku X-ray diffractometer with a Cu Kα radiation source (λ = 0.15406 nm) at a power of 1600 W (40 kV, 40 mA) over a 2θ range of 3˚– 40˚ at step size of 0.02˚ and scan speed of 0.2˚/min. Nitrogen sorption isotherms were measured at 77 K with a Microtrac BELSORP MINI X surface area and pore size distribution analyzer after the COF sample was degassed overnight at 120 °C under dynamic vacuum. The Brunauer−Emmett−Teller (BET) method was employed to evaluate the specific surface areas. The pore size distributions were derived from nitrogen adsorption isotherms by using the non-local density functional theory (NLDFT) in BELMaster7 analysis software. Low-pressure H_2, CO_2 and CH_4 adsorption measurements were conducted on a PhysiChem iPore 400 Automated Surface Area and Pore Size Analyzer. Liquid-state NMR spectra and solid-state ^{13}C cross-polarization magic-angle-spinning (CP/MAS) NMR spectra were collected on a Bruker-Biospin Avance Neo 400 NMR spectrometer. A 4 mm VTN probe was used and the spinning frequency was 8 kHz in the ^{13}C CP/MAS NMR experiment. Fourier transform infrared (FT-IR) spectra of building blocks and COF samples were acquired using a JASCO FT/IR-4600 FT-IR spectrometer over the wave number range of 4000 to 400 cm^{-1}. Thermogravimetric analysis (TGA) of TUS-84 was carried out on a Bruker AXS MS9610/DSC3200A/TG-DTA2010SA thermal analysis system after placing the sample in an alumina pan followed by heating from room temperature to 800 °C at a ramp rate of 10 °C min^{-1} under a nitrogen purge (100 mL min^{-}

[1]). Scanning electron microscopy (SEM) images were recorded at an accelerating voltage of 10 kV on a JEOL JSM-7000FSHL field emission scanning electron microscope. The TUS-84 COF sample was sputter coated with a very thin Au layer by a Sanyu Electron SC-701MkII ADVANCE sputtering equipment before obtaining SEM images. High-resolution transmission electron microscopy (HRTEM) investigation and corresponding fast Fourier transform (FFT) analysis was performed on a JEOL JEM-2100F microscope. The C, H and N contents in the COF sample was determined using a PerkinElmer 2400 Series II CHNS/O Elemental Analyzer.

2. Synthetic procedures

2.1. Synthesis of building blocks

4',5'-bis(3,5-diformylphenyl)-3',6'-dimethyl-[1,1':2',1"-terphenyl]-3,3",5,5"-tetracarbaldehyde (DPTB-Me)[1] and 5,10,15,20-tetrakis(4-aminophenyl)porphyrin (TAPP)[2-4] were synthesized according to the reported methods.

2.2. Synthesis of TUS-84

DPTB-Me (19.0 mg, 0.03 mmol) and **TAPP** (40.48 mg, 0.06 mmol) were introduced into a 10 mL Pyrex tube (body length = 14.5 cm, neck length = 9 cm), followed by the addition of 0.5 mL of mesitylene, 0.5 mL of 1,4-dioxane and 0.2 mL of 6 M aqueous acetic acid. The Pyrex tube was partially immersed in an ultrasonic bath for 15 minutes to produce a homogenous dispersion. After this, the tube was degassed by three freeze-pump-thaw cycles and flame sealed under vacuum. The sealed tube was left for aging at ambient temperature for 3 hours and then placed in an oven at 120 °C for 3 days. The resulting precipitate was isolated by centrifugation and washed several times with tetrahydrofuran, until a clear supernatant could be obtained. The solid was left to dry overnight at room temperature, followed by washing with tetrahydrofuran in a Soxhlet extractor for 24 hours. The solid product was activated under dynamic vacuum at room temperature for 5 hours followed by dynamic vacuum at 100 °C for 3 hours to afford a dark purple powder of activated TUS-84 (38.75 mg). Anal. Calcd. for $C_{512}H_{272}N_{64}$: C: 83.76; H: 4.08; N: 12.16. Found: C: 86.91; H: 4.6; N: 8.49.

3. Solid-state ^{13}C CP/MAS Nuclear Magnetic Resonance Spectroscopy

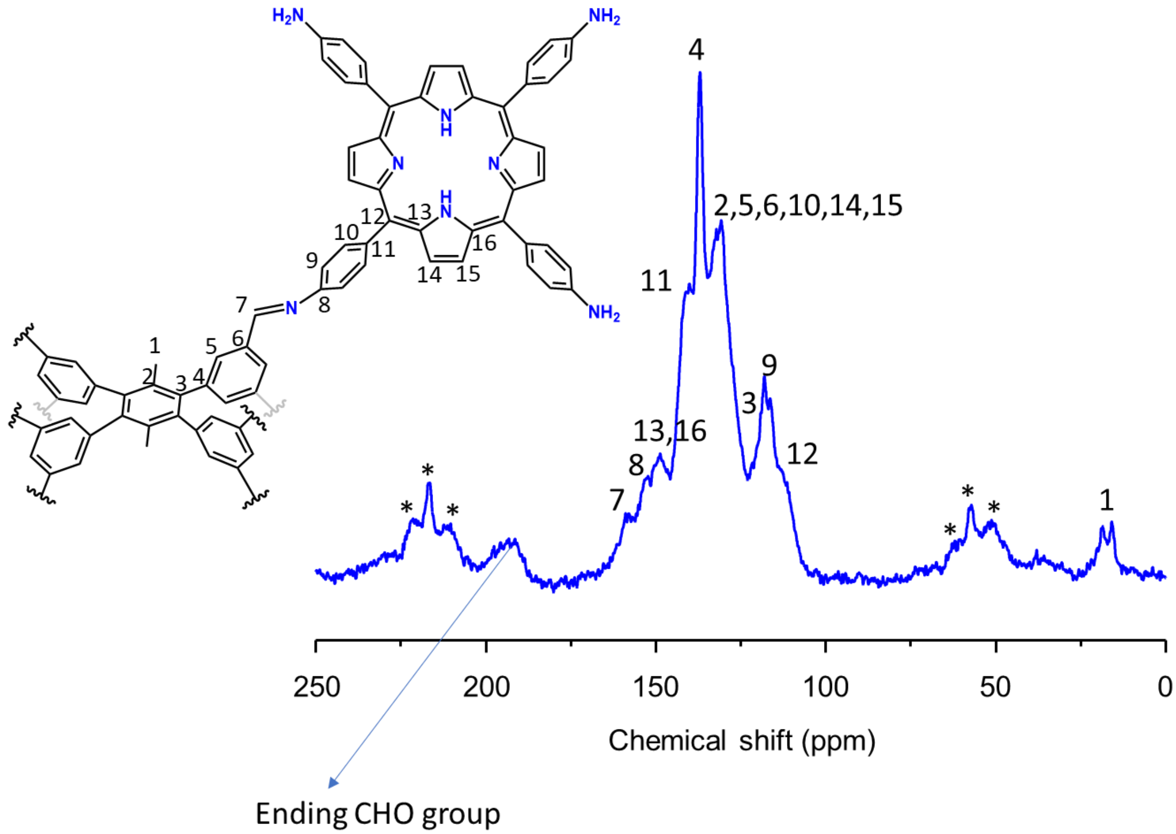

Figure S1. Solid-state ^{13}C CP/MAS NMR spectrum of TUS-84. The asterisk denotes spinning sideband.

4. Fourier-Transform Infrared (FT-IR) Spectroscopy

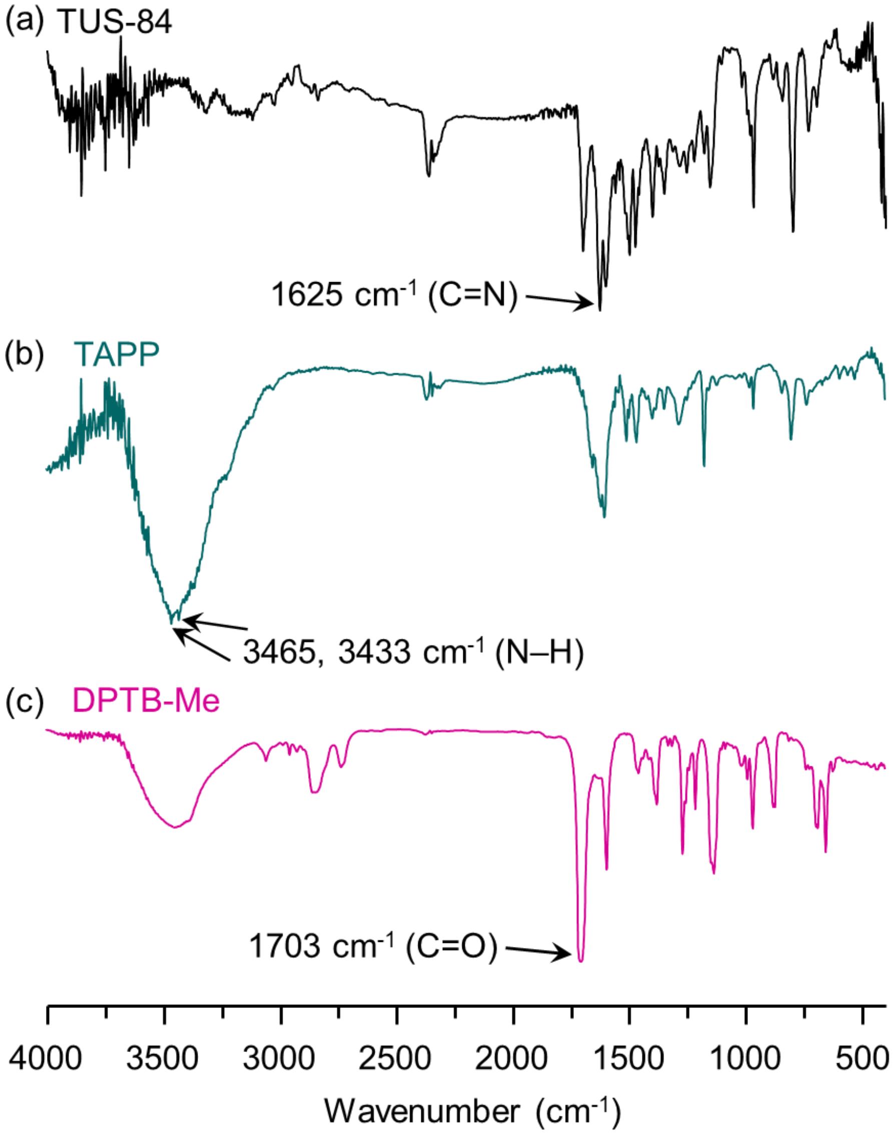

Figure S2. FT-IR spectra of (a) TUS-84, (b) TAPP, and (c) DPTB-Me.

5. Scanning electron microscopy (SEM) and Transmission electron microscopy (TEM) images

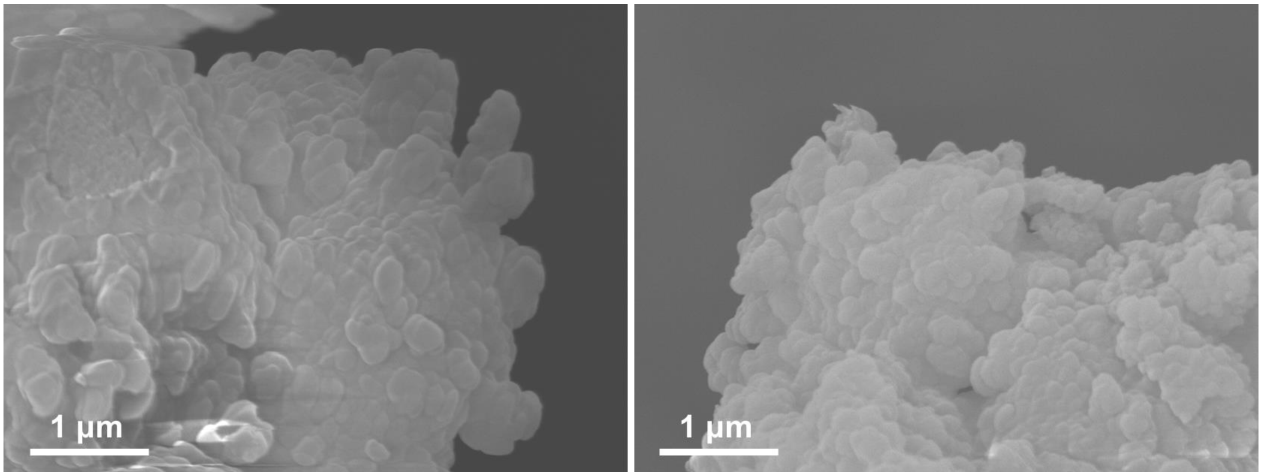

Figure S3. SEM images of TUS-84.

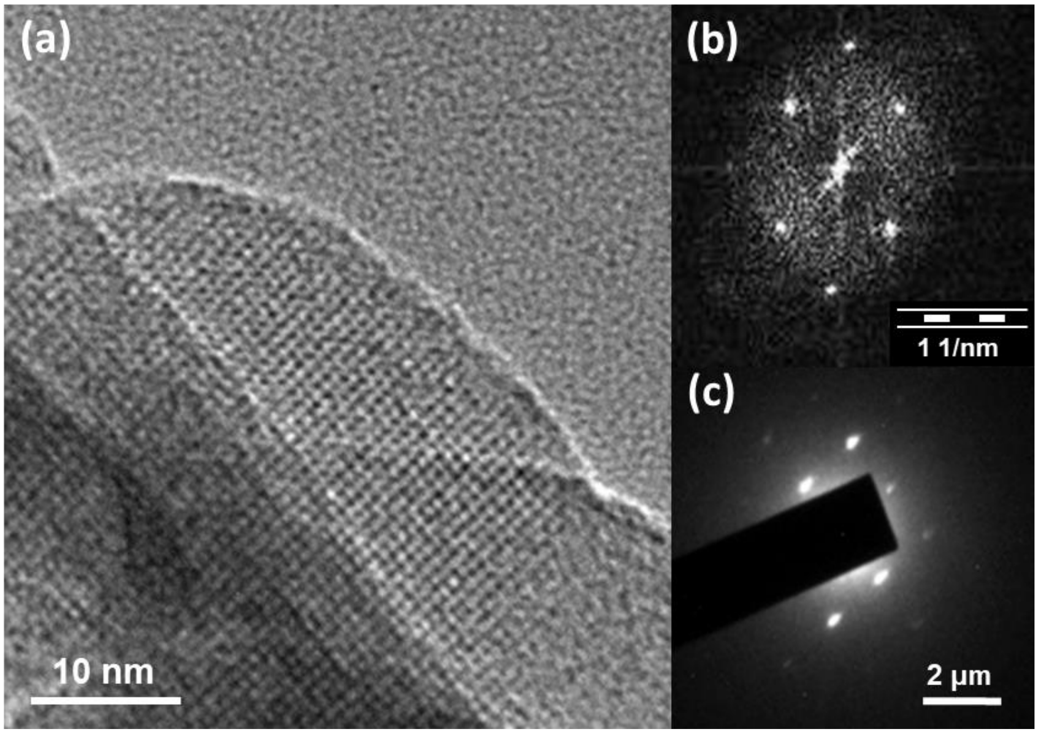

Figure S4. (a) HRTEM image of TUS-84. (b) Fast Fourier transform (FFT) pattern and (c) selected area electron diffraction (SAED) pattern acquired from the area shown in Figure a.

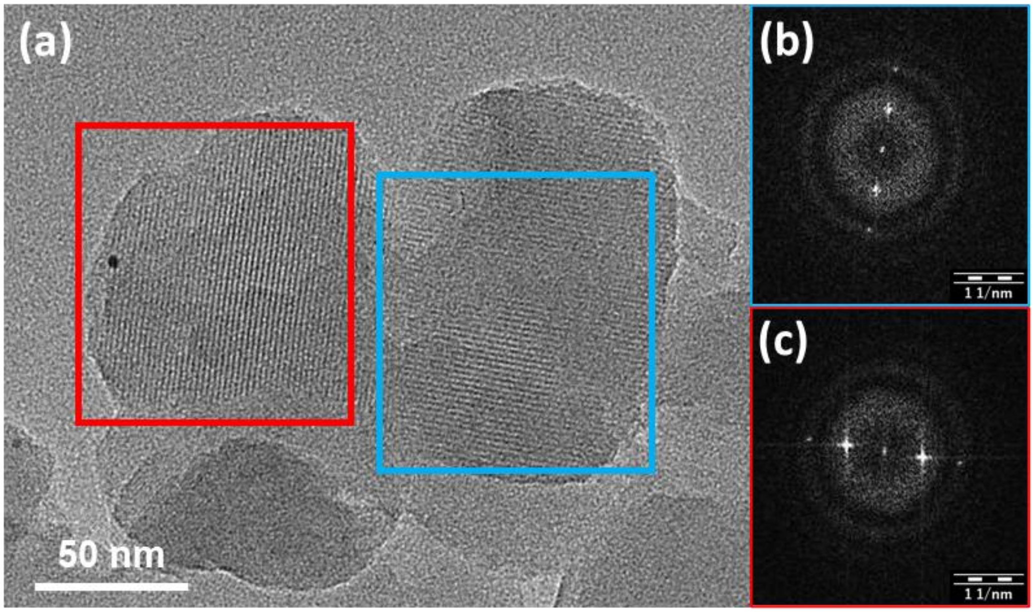

Figure S5. (a) HRTEM image of TUS-84. (b,c) Fast Fourier transform (FFT) patterns acquired from the areas enclosed by the boxes marked in corresponding colors.

6. Thermogravimetric analysis (TGA)

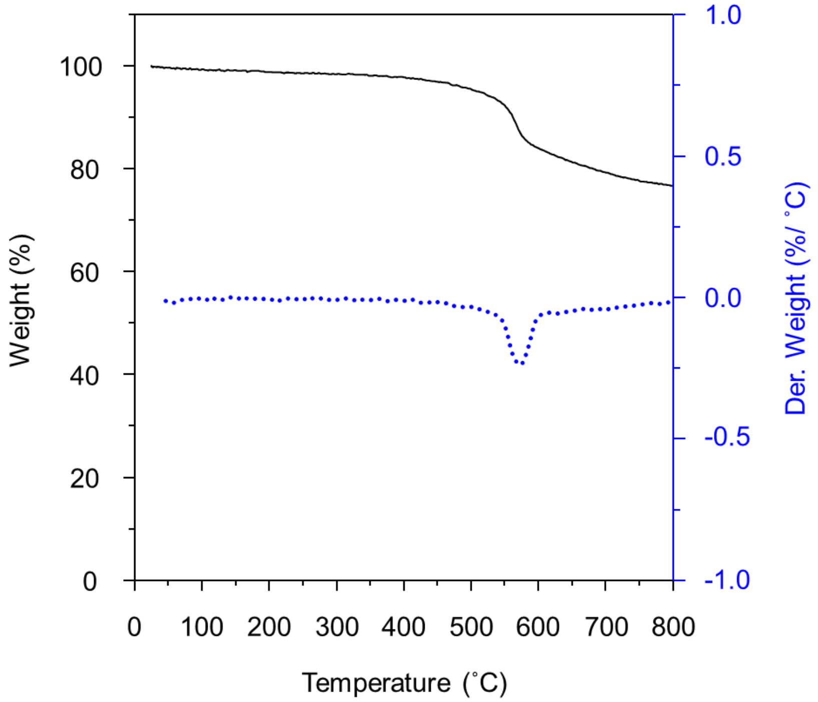

Figure S6. TGA trace of TUS-84 under N_2 atmosphere.

7. Chemical stability test

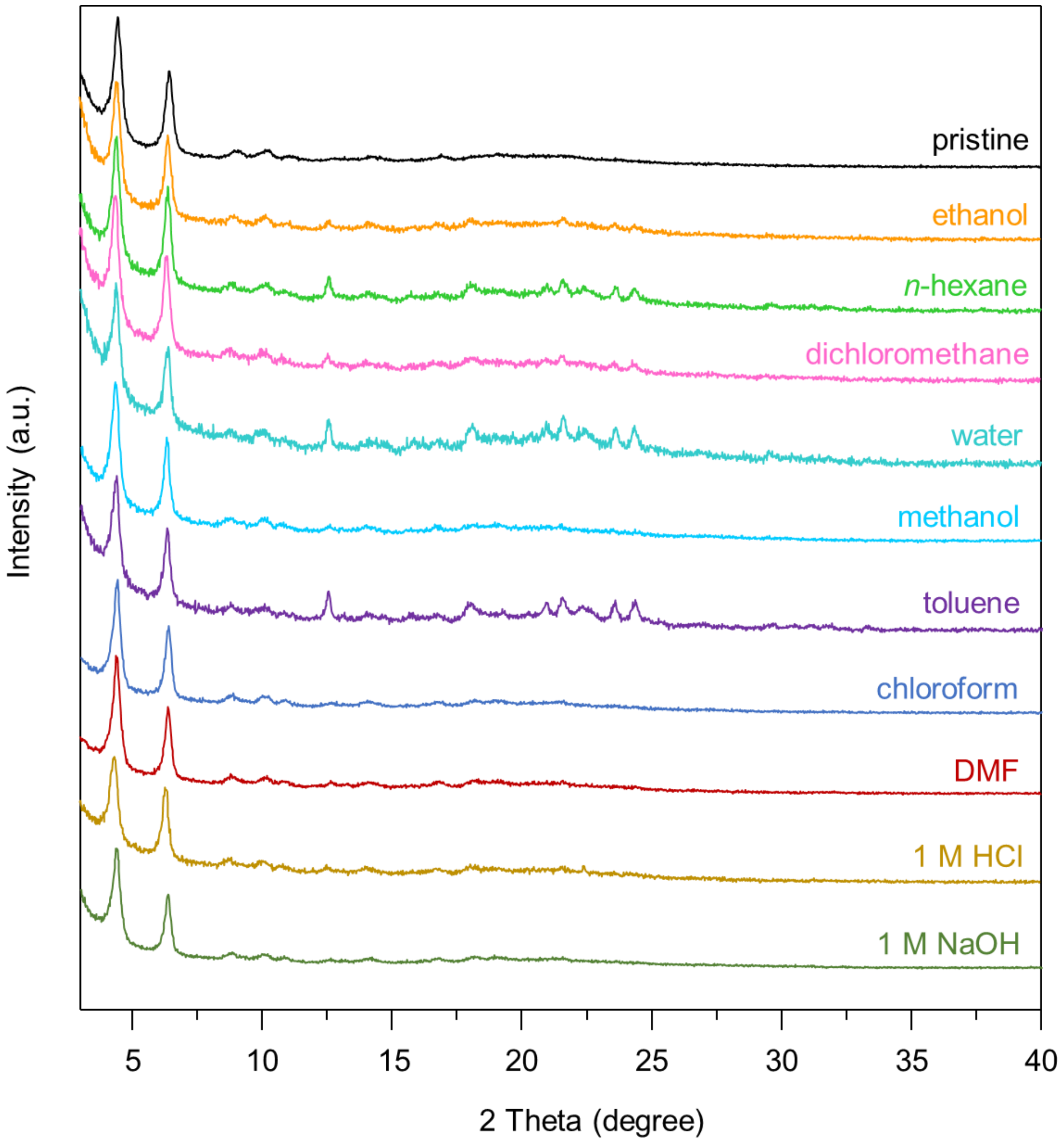

Figure S7. PXRD profiles of TUS-84, pristine and after treatment in different solvents.

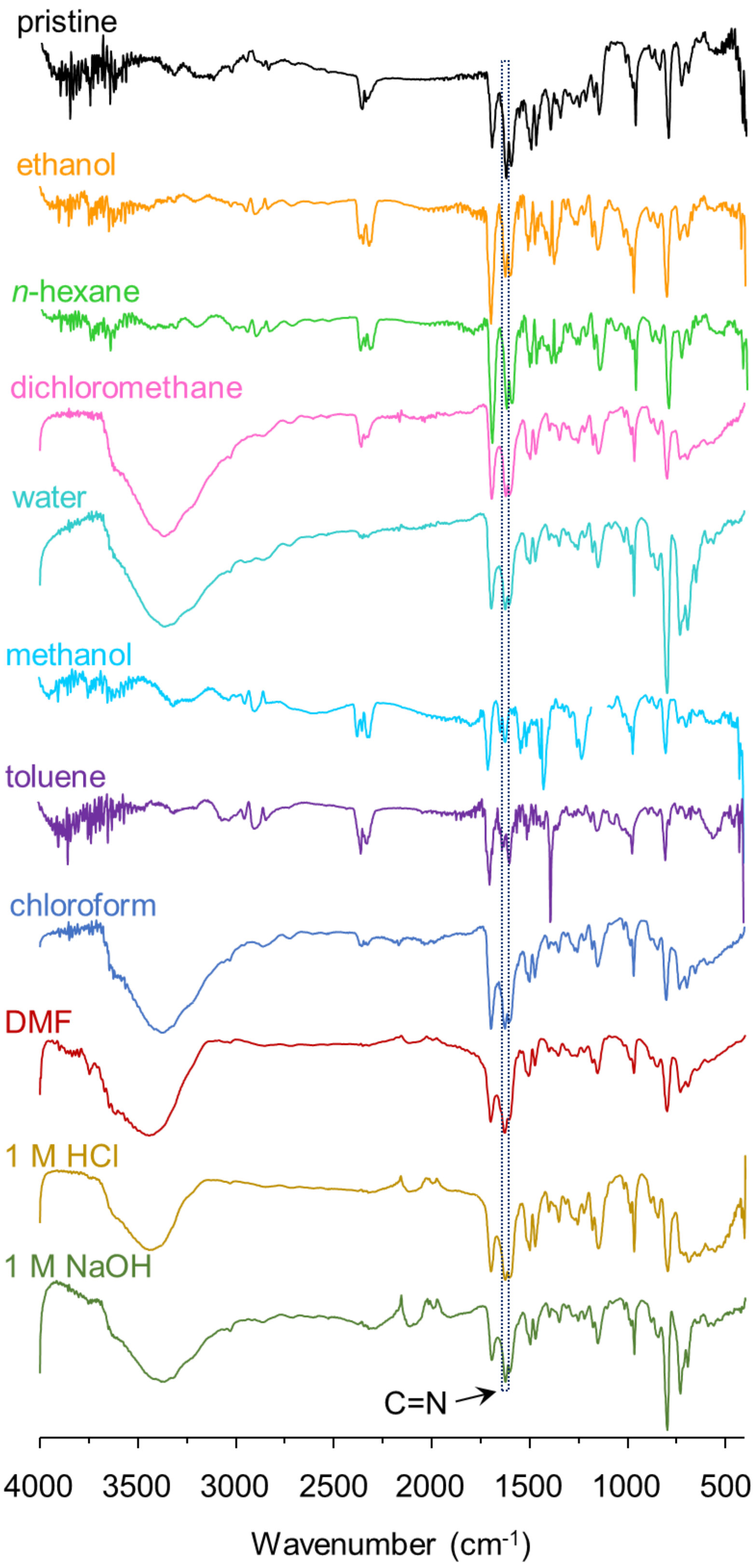

Figure S8. FT-IR spectra of TUS-84, pristine and after treatment in different solvents.

8. N_2 adsorption

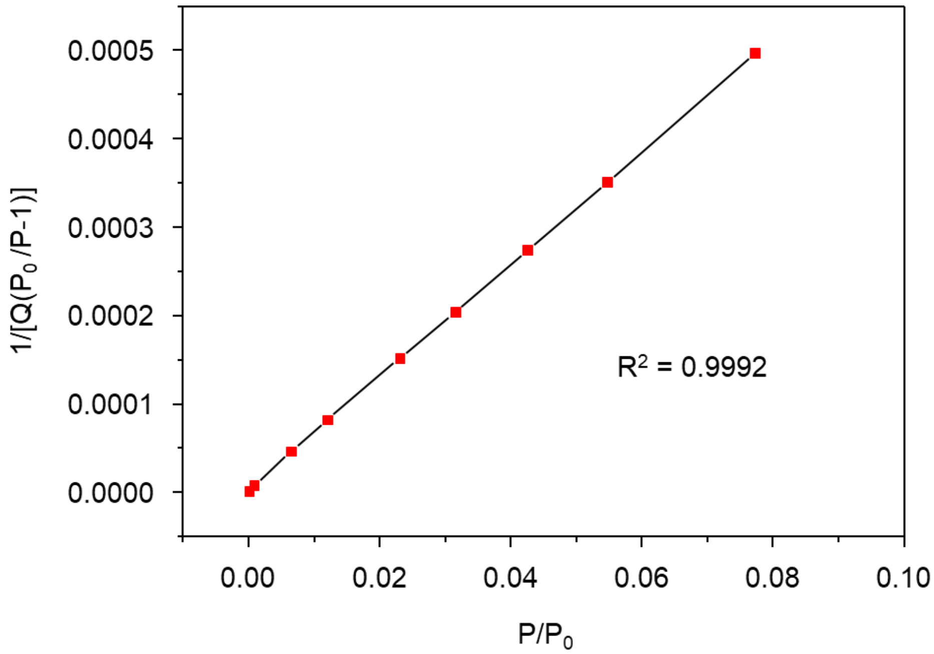

Figure S9. BET surface area plot for TUS-84 calculated from the N_2 adsorption isotherms at 77 K.

9. H_2, CO_2 and CH_4 adsorption

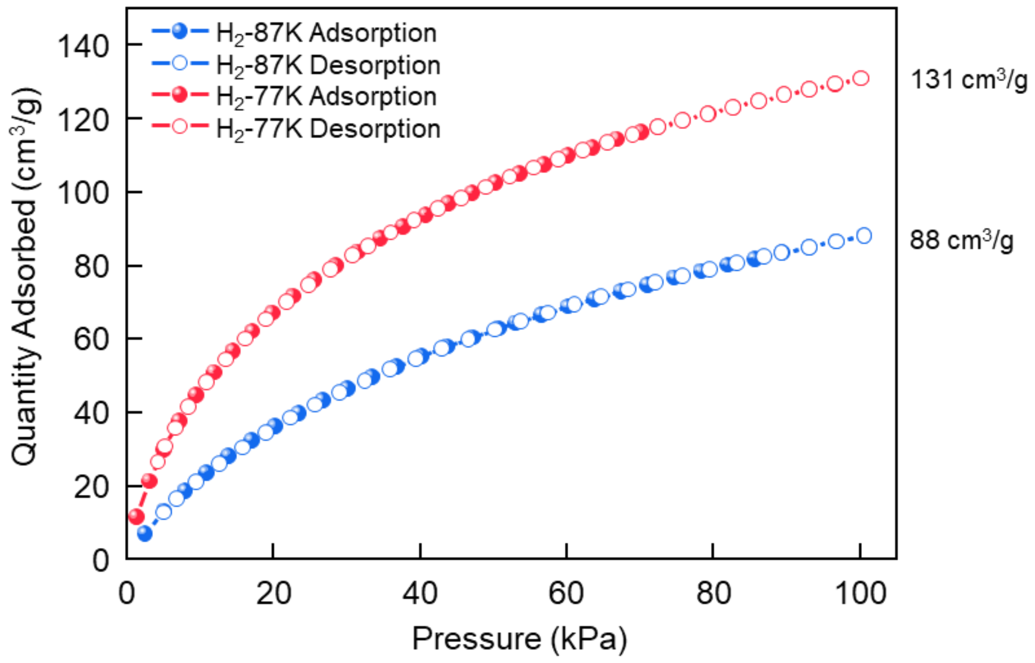

Figure S10. H_2 sorption isotherms of TUS-84.

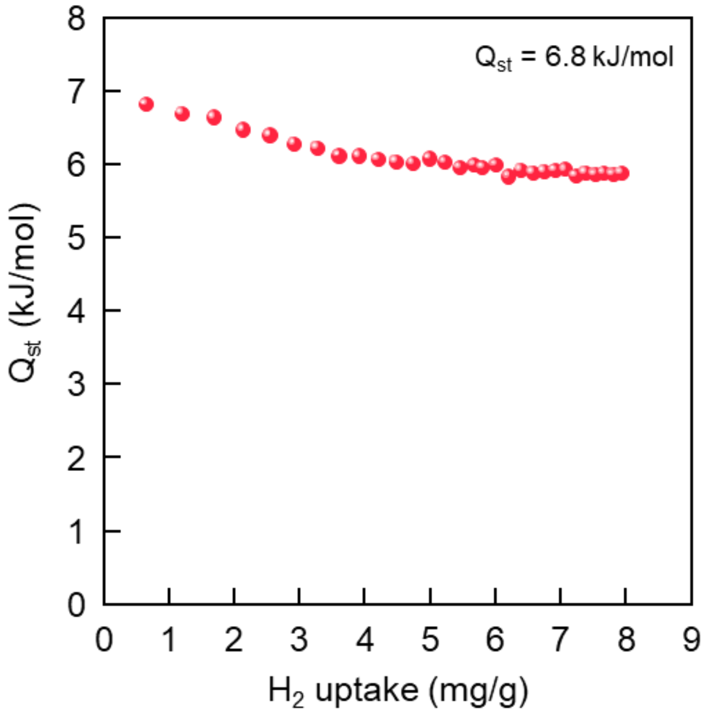

Figure S11. Isosteric enthalpies of adsorption (Q_{st}) for H_2, evaluated using the Clausius–Clapeyron equation.

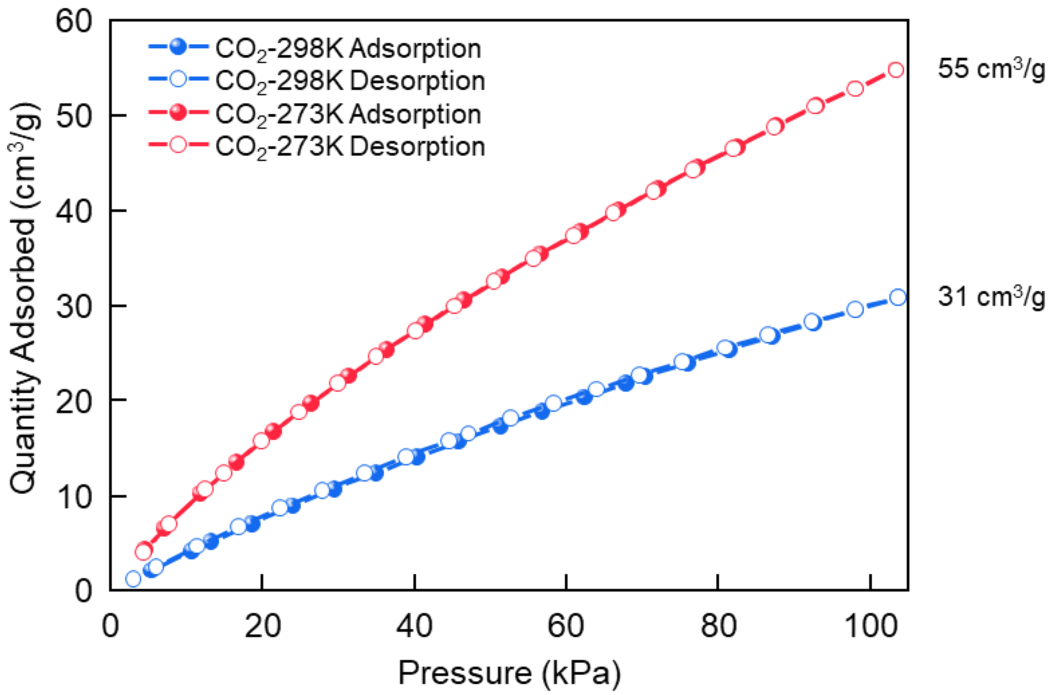

Figure S12. CO_2 sorption isotherms of TUS-84.

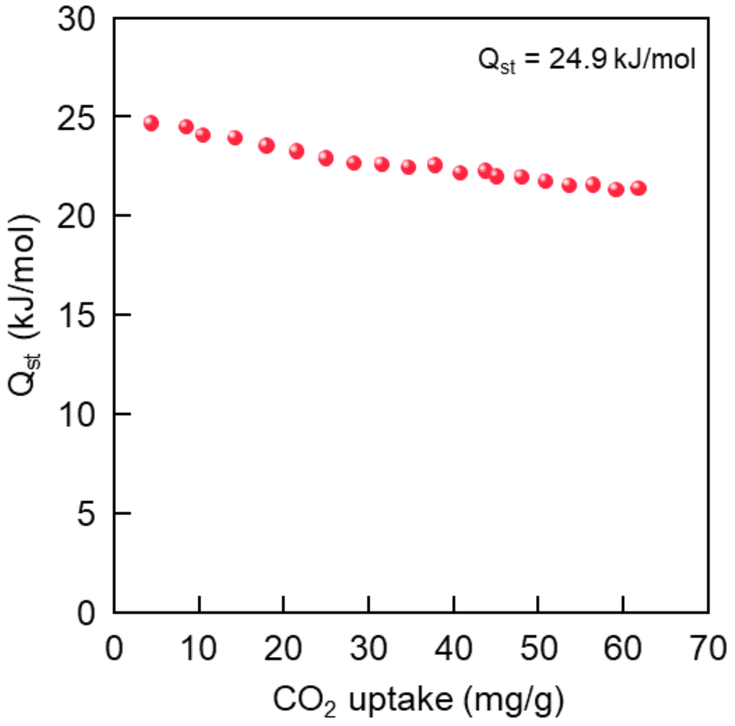

Figure S13. Isosteric enthalpies of adsorption (Q_{st}) for CO_2, evaluated using the Clausius–Clapeyron equation.

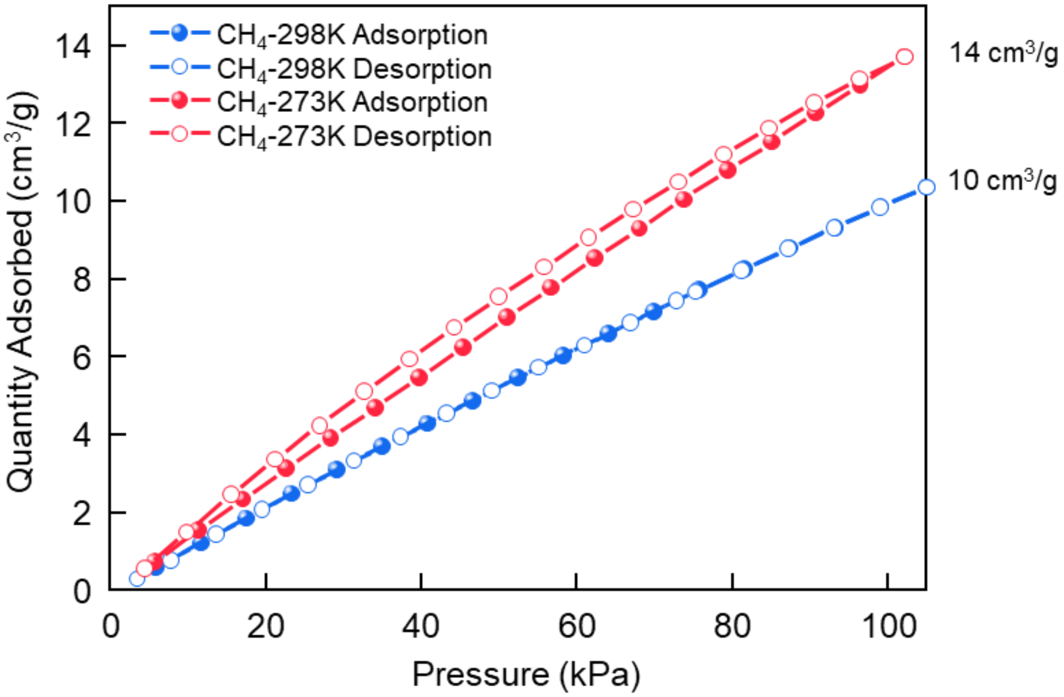

Figure S14. CH_4 sorption isotherms of TUS-84.

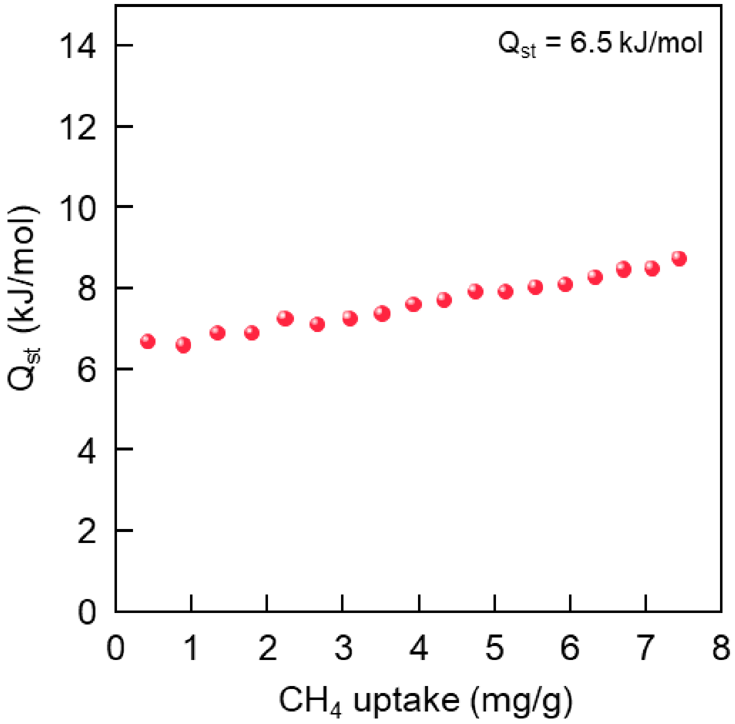

Figure S15. Isosteric enthalpies of adsorption (Q_{st}) for CH_4, evaluated using the Clausius–Clapeyron equation.

10. Structure simulations and X-ray diffraction analyses

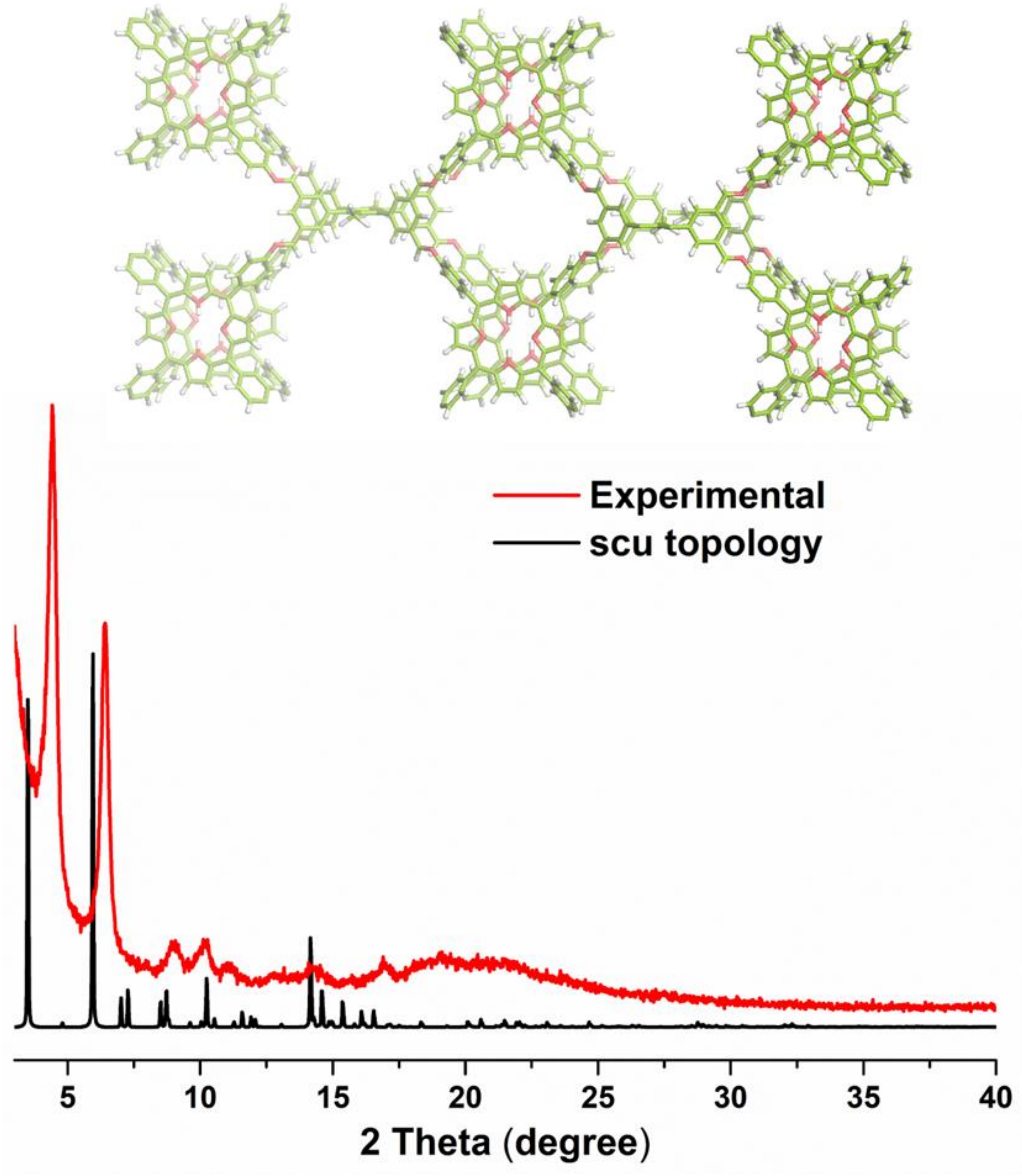

Figure S16. Calculated XRD pattern and stick model of TUS-84 based on the non-interpenetrated **scu** net.

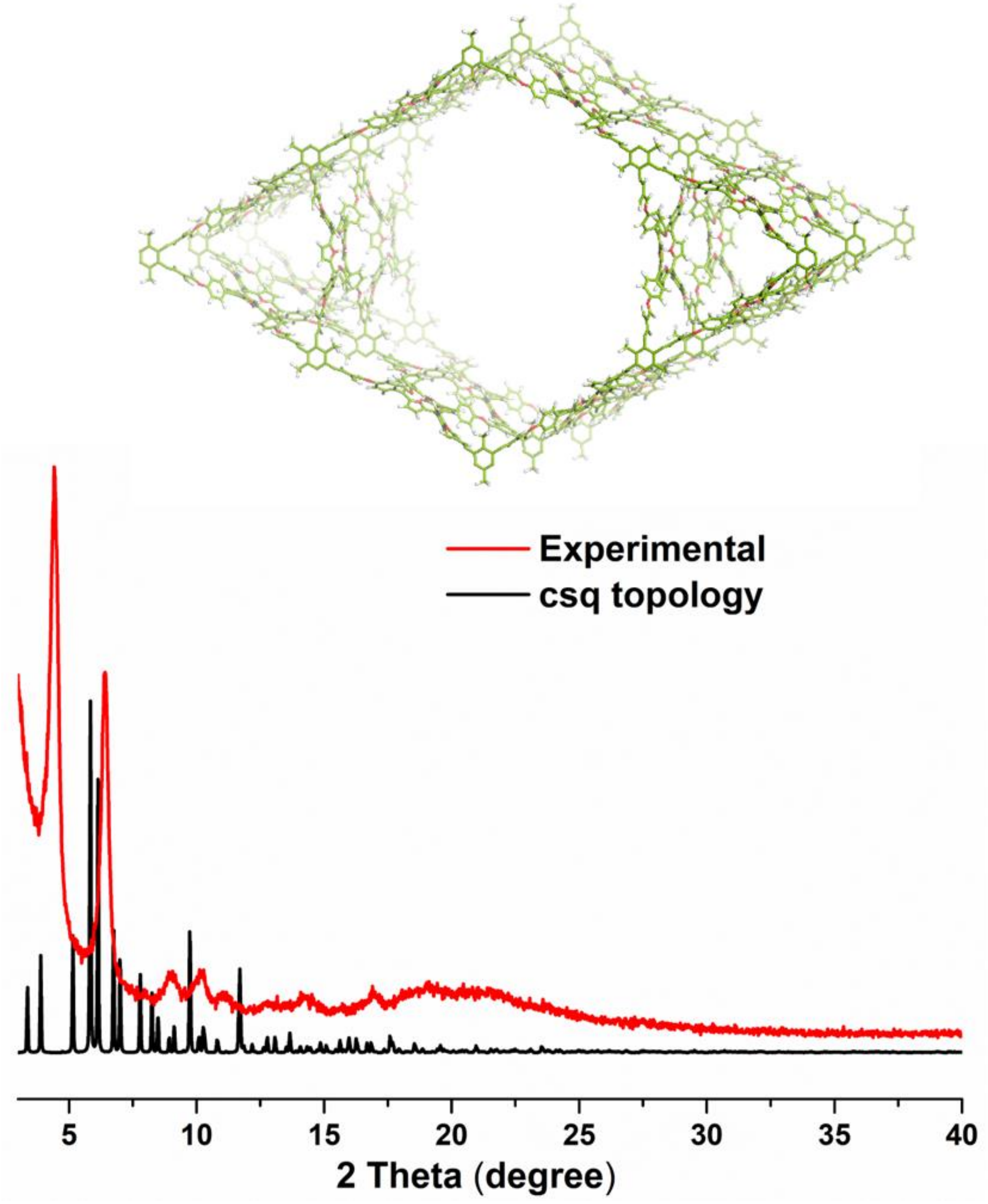

Figure S17. Calculated XRD pattern and stick model of TUS-84 based on the **csq** net.

11. Drug delivery

11.1. Loading and release of Ibuprofen

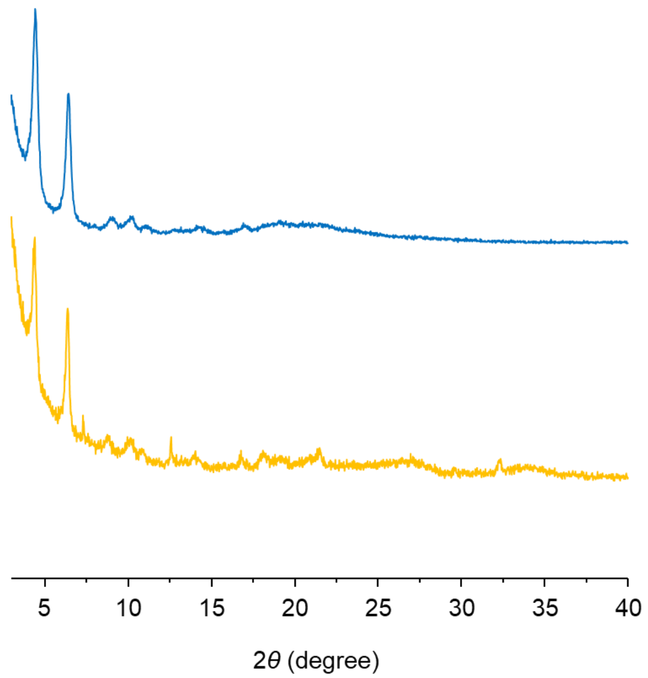

Figure S18. PXRD pattern of TUS-84 (blue curve) and ibuprofen-loaded TUS-84 (yellow curve).

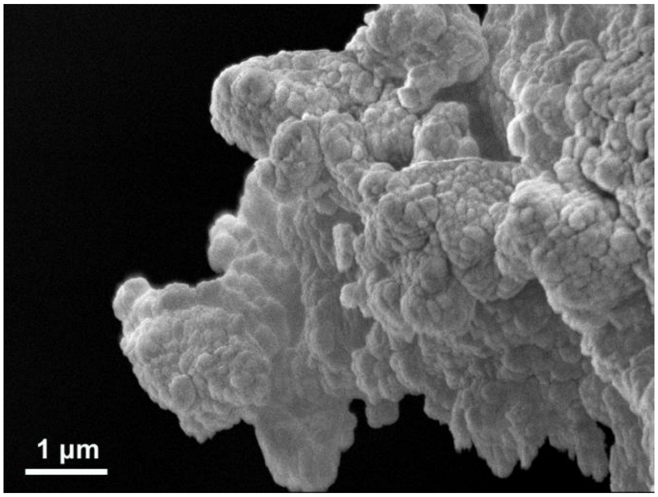

Figure S19. SEM image of ibuprofen-loaded TUS-84.

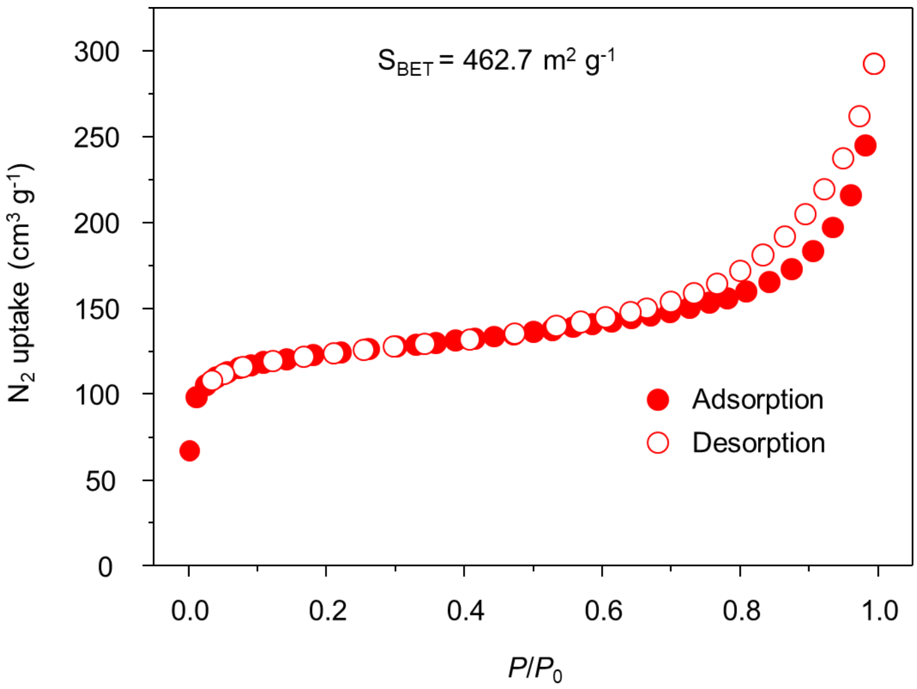

Figure S20. Nitrogen sorption isotherms of ibuprofen-loaded TUS-84

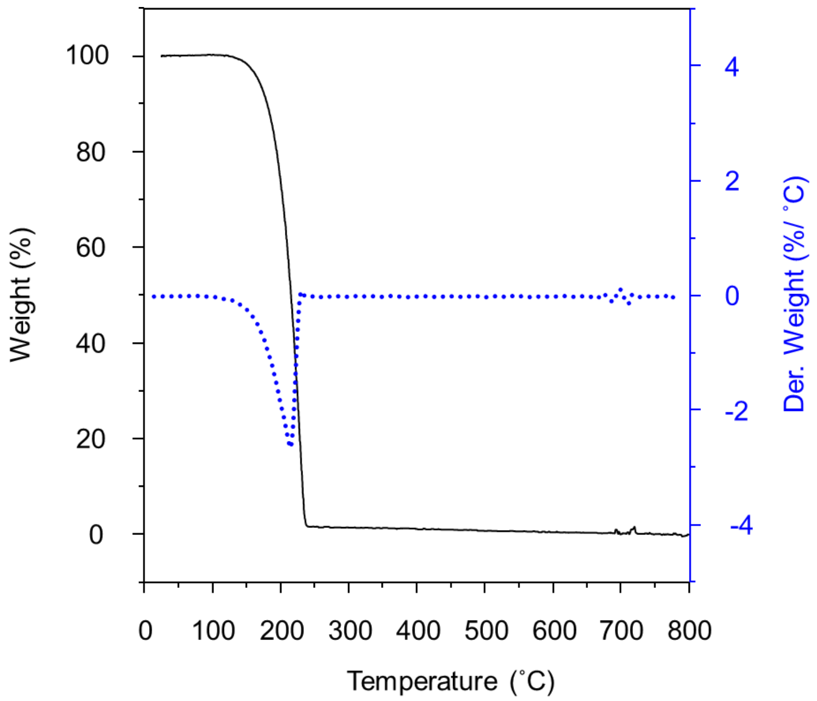

Figure S21. TGA trace of ibuprofen under N_2 atmosphere.

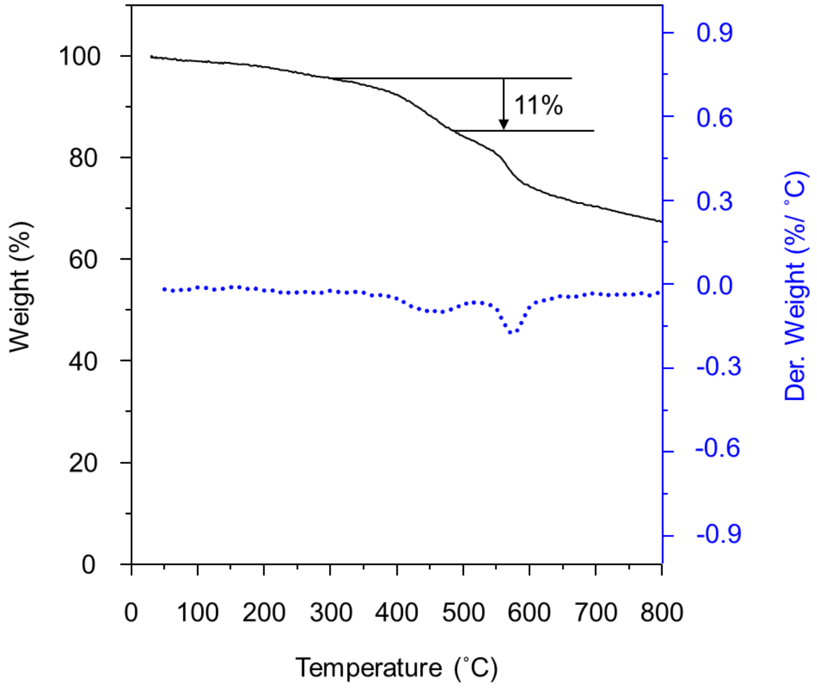

Figure S22. TGA trace of ibuprofen-loaded TUS-84 under N_2 atmosphere.

Evaluation of ibuprofen loading from UV-vis absorption

Step 1:

618.8 mg (3 mmol) of ibuprofen was dissolved in **30 mL** of n-hexane.

51.5 mg of COF was added to this solution.

Step 2:

After 50 times dilution, the supernatant UV-vis absorbance:

Before loading: 0.42072003 (After 50 times dilution)

After 4 h loading: 0.416418189 (After 50 times dilution)

Step 3:

From the calibration curve,

y = 0.208x + 0.0242, R^2 = 0.999

where y = absorbance, x = concentration of ibuprofen (mM)

Step 4:

Concentration (Calculation from curve)

Before loading (C_1): **95.317 mM**

After 4 h loading (C_2): **94.283 mM**

Step 5:

Loading weight of ibuprofen (mg)

$$= \frac{(C_1 - C_2) \times \text{Amount of hexane (mL)} \times \text{Molecular weight of ibuprofen (g mol}^{-1}\text{)}}{1000}$$

$$= \frac{(95.317 - 94.283) \times 30 \times 206.28}{1000}$$

= **6.39 mg**

Step 6:

Loading weight of ibuprofen (%)

$$= \frac{100 \times 6.3988}{(51.5+6.3988)}$$

= **11.05 wt%**

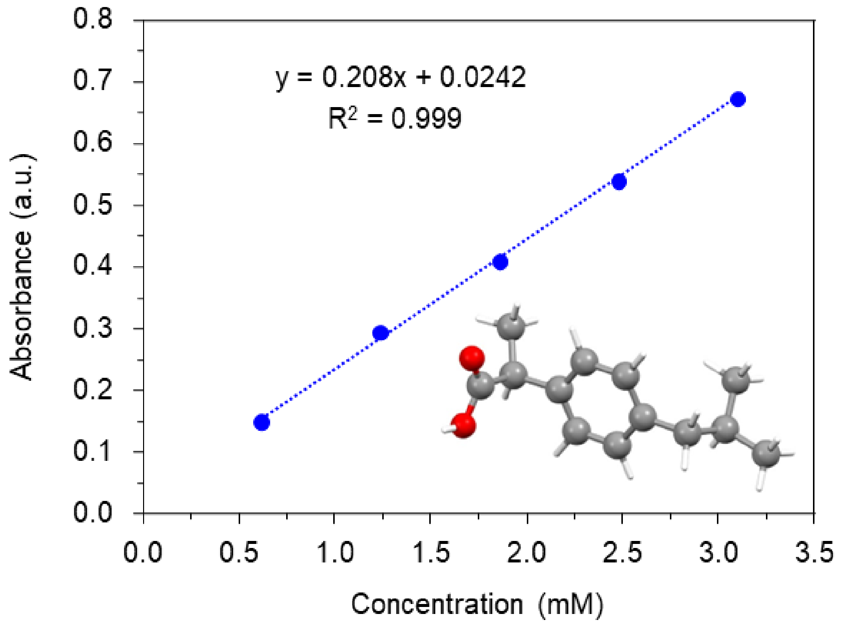

Figure S23. Calibration curve of ibuprofen in n-hexane. The intensities were recorded at 261 nm. Inset shows the chemical structure of ibuprofen (C: gray, H: white, O: red).

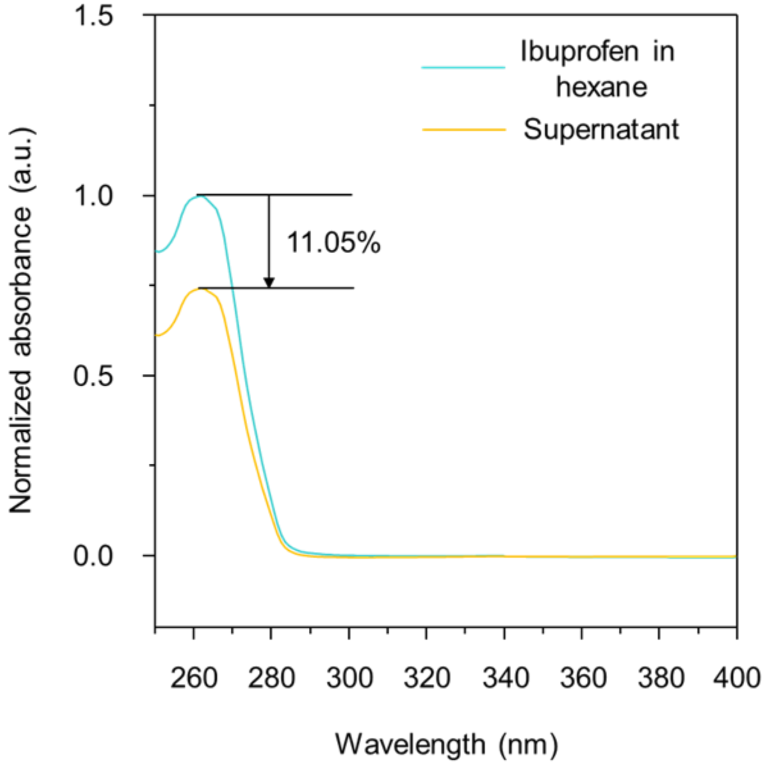

Figure S24. Evaluation of drug loading from UV-vis spectrophotometry.

11.1. Loading and release of Captopril

For loading of captopril, 50 mg of TUS-84 was suspended in 30 mL of 0.1 M aqueous solution of captopril under magnetic stirring for 4 h.

The captopril release study was performed by placing 40 mg of the captopril-loaded TUS-84 sample inside a semipermeable bag followed by immersing in 10 mL of phosphate buffer solution (simulated body fluid, pH 7.4) at a constant temperature of 37 °C.

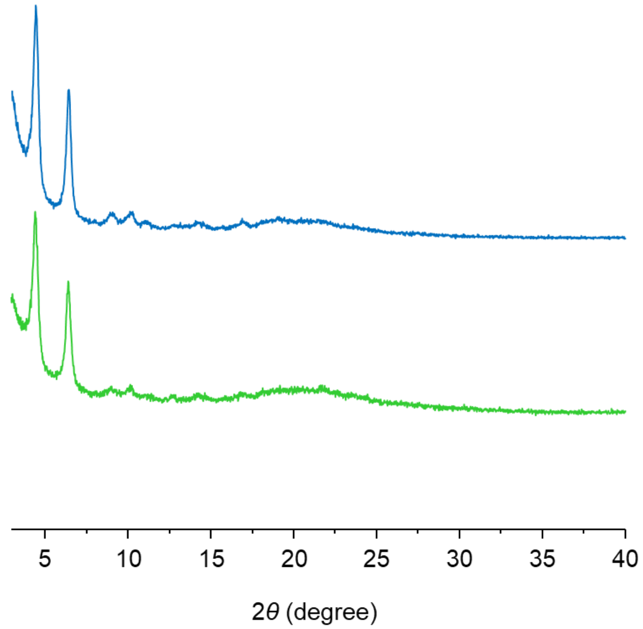

Figure S25. PXRD pattern of TUS-84 (blue curve) and captopril-loaded TUS-84 (green curve).

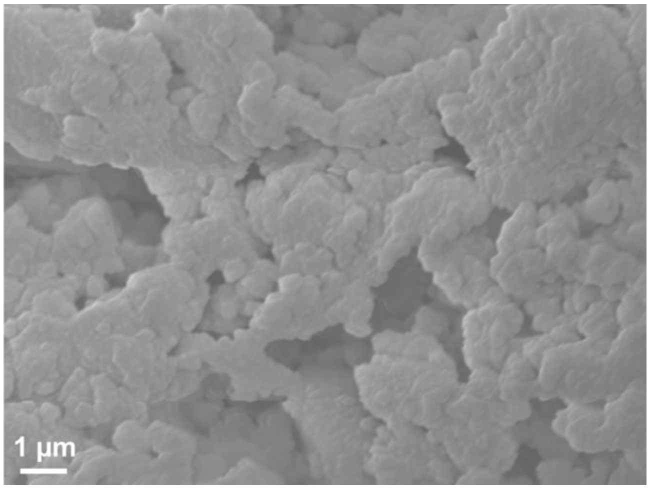

Figure S26. SEM image of captopril-loaded TUS-84.

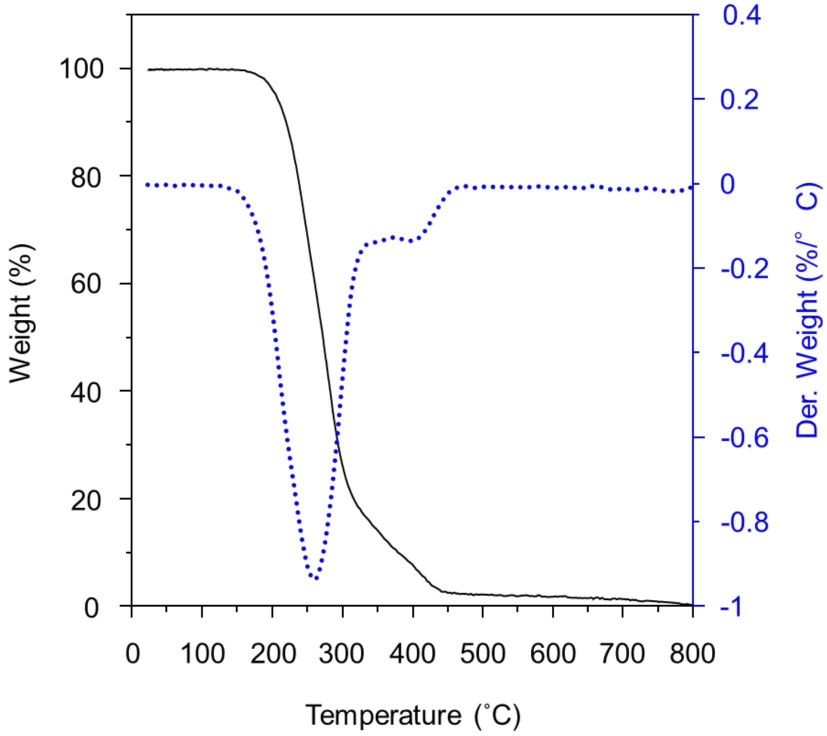

Figure S27. TGA trace of captopril under N_2 atmosphere.

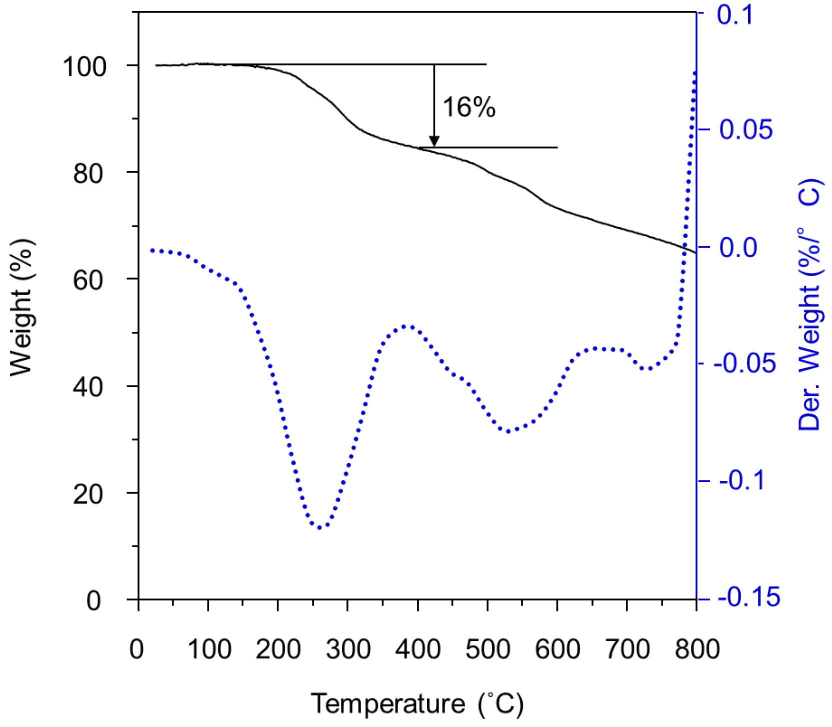

Figure S28. TGA trace of captopril-loaded TUS-84 under N_2 atmosphere.

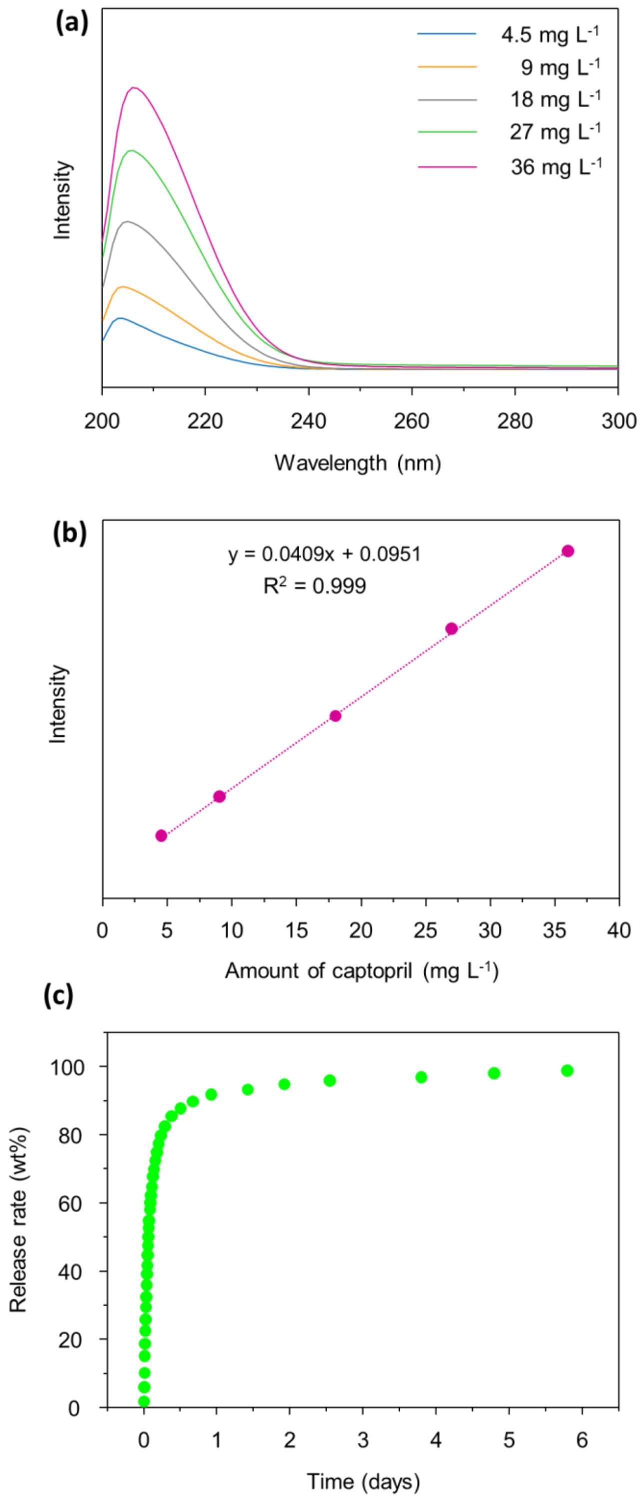

Figure 29. (a) UV-Vis spectra of captopril in simulated body fluid (pH 7.4, phosphate buffer solution) at different concentrations. (b) Calibration curve of captopril. (c) Release profile of captopril from captopril-loaded TUS-84.

12. Crystallographic information

Table S1. Fractional atomic coordinates for the unit cell of TUS-84 calculated based on the **scu-c** net.

Space group		*Pm*	
Calculated unit cell		a = 39.9205 Å, b = 18.7162 Å, c = 23.6564 Å, $\alpha = \beta = \gamma = 90°$	
Measured unit cell		a = 39.9179 Å, b = 18.7054 Å, c = 23.6772 Å, $\alpha = \beta = \gamma = 90°$	
Pawley refinement		R_p = 4.37 %, R_{wp} = 3.19 %	
atoms	x	y	z
C1	0.18639	0.83862	0.04937
C2	0.15761	0.88063	0.03186
C3	0.18847	0.89333	0.21396
C4	0.17036	0.90292	0.26897
C5	0.3785	0.15972	0.27825
C6	0.35997	0.17128	0.33302
C7	0.84238	0.61937	0.46814
C8	0.76478	0.72959	0.31424
C9	0.22654	0.21679	0.12569
C10	0.23522	0.22959	0.18576
C11	0.14003	0.17128	0.16698
C12	0.1215	0.15972	0.22175
C13	0.82964	0.40292	0.23103
C14	0.81153	0.39333	0.28604
C15	0.19637	0.82537	0.11059
C16	0.13697	0.8746	0.27382
C17	0.17406	0.8535	0.16291
C18	0.1343	0.87742	0.38479
N19	0.11892	0.87771	0.3315
C20	0.08107	0.9041	0.444
C21	0.11548	0.88289	0.44355
C22	0.38452	0.11712	0.05645
C23	0.41893	0.0959	0.056
N24	0.38108	0.1223	0.1685
C25	0.3657	0.12259	0.11521
C26	0.32594	0.1465	0.33709
C27	0.36303	0.1254	0.22618
C28	0.30363	0.17463	0.38941
C29	0.27346	0.21679	0.37431
C30	0.31361	0.16138	0.45063

H31	0.13994	0.90644	0.06859
H32	0.21539	0.91795	0.20984
H33	0.18274	0.93392	0.31022
H34	0.40634	0.1783	0.27588
H35	0.37254	0.20098	0.37466
H36	0.86006	0.59356	0.43141
H37	0.7774	0.71279	0.26856
H38	0.21815	0.20654	0.22448
H39	0.12746	0.20098	0.12534
H40	0.09366	0.1783	0.22412
H41	0.81726	0.43392	0.18978
H42	0.78461	0.41795	0.29016
H43	0.16319	0.87277	0.38515
H44	0.0669	0.91294	0.39851
H45	0.4331	0.08706	0.10149
H46	0.33681	0.12725	0.11485
C47	0.68639	0.33862	0.04937
C48	0.65761	0.38063	0.03186
C49	0.68847	0.39333	0.21396
C50	0.67036	0.40292	0.26897
C51	0.8785	0.65972	0.27825
C52	0.85997	0.67128	0.33302
C53	0.34238	0.11937	0.46814
C54	0.26478	0.22959	0.31424
C55	0.72654	0.71679	0.12569
C56	0.73522	0.72959	0.18576
C57	0.64003	0.67128	0.16698
C58	0.6215	0.65972	0.22175
C59	0.32964	0.90292	0.23103
C60	0.31153	0.89333	0.28604
C61	0.69637	0.32537	0.11059
C62	0.63697	0.3746	0.27382
C63	0.67406	0.3535	0.16291
C64	0.6343	0.37742	0.38479
N65	0.61892	0.37771	0.3315
C66	0.58107	0.4041	0.444
C67	0.61548	0.38289	0.44355
C68	0.88452	0.61712	0.05645
C69	0.91893	0.5959	0.056
N70	0.88108	0.6223	0.1685
C71	0.8657	0.62259	0.11521
C72	0.82594	0.6465	0.33709
C73	0.86303	0.6254	0.22618

C74	0.80363	0.67463	0.38941
C75	0.77346	0.71679	0.37431
C76	0.81361	0.66138	0.45063
H77	0.63994	0.40644	0.06859
H78	0.71539	0.41795	0.20984
H79	0.68274	0.43392	0.31022
H80	0.90634	0.6783	0.27588
H81	0.87254	0.70098	0.37466
H82	0.36006	0.09356	0.43141
H83	0.2774	0.21279	0.26856
H84	0.71815	0.70654	0.22448
H85	0.62746	0.70098	0.12534
H86	0.59366	0.6783	0.22412
H87	0.31726	0.93392	0.18978
H88	0.28461	0.91795	0.29016
H89	0.66319	0.37277	0.38515
H90	0.5669	0.41294	0.39851
H91	0.9331	0.58706	0.10149
H92	0.83681	0.62725	0.11485
C93	0.81361	0.16138	0.04937
C94	0.84239	0.11937	0.03186
C95	0.81153	0.10667	0.21396
C96	0.82964	0.09708	0.26897
C97	0.6215	0.84028	0.27825
C98	0.64003	0.82872	0.33302
C99	0.15762	0.38063	0.46814
C100	0.23522	0.27041	0.31424
C101	0.77346	0.78321	0.12569
C102	0.76478	0.77041	0.18576
C103	0.85997	0.82872	0.16698
C104	0.8785	0.84028	0.22175
C105	0.17036	0.59708	0.23103
C106	0.18847	0.60667	0.28604
C107	0.80363	0.17463	0.11059
C108	0.86303	0.1254	0.27382
C109	0.82594	0.1465	0.16291
C110	0.8657	0.12258	0.38479
N111	0.88108	0.12229	0.3315
C112	0.91893	0.0959	0.444
C113	0.88452	0.11711	0.44355
C114	0.61548	0.88288	0.05645
C115	0.58107	0.9041	0.056
N116	0.61892	0.8777	0.1685

C117	0.6343	0.87741	0.11521
C118	0.67406	0.8535	0.33709
C119	0.63697	0.8746	0.22618
C120	0.69637	0.82537	0.38941
C121	0.72654	0.78321	0.37431
C122	0.68639	0.83862	0.45063
H123	0.86006	0.09356	0.06859
H124	0.78461	0.08205	0.20984
H125	0.81726	0.06608	0.31022
H126	0.59366	0.8217	0.27588
H127	0.62746	0.79902	0.37466
H128	0.13994	0.40644	0.43141
H129	0.2226	0.28721	0.26856
H130	0.78185	0.79346	0.22448
H131	0.87254	0.79902	0.12534
H132	0.90634	0.8217	0.22412
H133	0.18274	0.56608	0.18978
H134	0.21539	0.58205	0.29016
H135	0.83681	0.12723	0.38515
H136	0.9331	0.08706	0.39851
H137	0.5669	0.91294	0.10149
H138	0.66319	0.87275	0.11485
C139	0.31361	0.66138	0.04937
C140	0.34239	0.61937	0.03186
C141	0.31153	0.60667	0.21396
C142	0.32964	0.59708	0.26897
C143	0.1215	0.34028	0.27825
C144	0.14003	0.32872	0.33302
C145	0.65762	0.88063	0.46814
C146	0.73522	0.77041	0.31424
C147	0.27346	0.28321	0.12569
C148	0.26478	0.27041	0.18576
C149	0.35997	0.32872	0.16698
C150	0.3785	0.34028	0.22175
C151	0.67036	0.09708	0.23103
C152	0.68847	0.10667	0.28604
C153	0.30363	0.67463	0.11059
C154	0.36303	0.6254	0.27382
C155	0.32594	0.6465	0.16291
C156	0.3657	0.62258	0.38479
N157	0.38108	0.62229	0.3315
C158	0.41893	0.5959	0.444
C159	0.38452	0.61711	0.44355

C160	0.11548	0.38288	0.05645
C161	0.08107	0.4041	0.056
N162	0.11892	0.3777	0.1685
C163	0.1343	0.37741	0.11521
C164	0.17406	0.3535	0.33709
C165	0.13697	0.3746	0.22618
C166	0.19637	0.32537	0.38941
C167	0.22654	0.28321	0.37431
C168	0.18639	0.33862	0.45063
H169	0.36006	0.59356	0.06859
H170	0.28461	0.58205	0.20984
H171	0.31726	0.56608	0.31022
H172	0.09366	0.3217	0.27588
H173	0.12746	0.29902	0.37466
H174	0.63994	0.90644	0.43141
H175	0.7226	0.78721	0.26856
H176	0.28185	0.29346	0.22448
H177	0.37254	0.29902	0.12534
H178	0.40634	0.3217	0.22412
H179	0.68274	0.06608	0.18978
H180	0.71539	0.08205	0.29016
H181	0.33681	0.62723	0.38515
H182	0.4331	0.58706	0.39851
H183	0.0669	0.41294	0.10149
H184	0.16319	0.37275	0.11485
C185	0.81361	0.83862	0.95063
C186	0.84239	0.88063	0.96814
C187	0.81153	0.89333	0.78604
C188	0.82964	0.90292	0.73103
C189	0.6215	0.15972	0.72175
C190	0.64003	0.17128	0.66698
C191	0.15762	0.61937	0.53186
C192	0.23522	0.72959	0.68576
C193	0.77346	0.21679	0.87431
C194	0.76478	0.22959	0.81424
C195	0.85997	0.17128	0.83302
C196	0.8785	0.15972	0.77825
C197	0.17036	0.40292	0.76897
C198	0.18847	0.39333	0.71396
C199	0.80363	0.82537	0.88941
C200	0.86303	0.8746	0.72618
C201	0.82594	0.8535	0.83709
C202	0.8657	0.87742	0.61521

N203	0.88108	0.87771	0.6685
C204	0.91893	0.9041	0.556
C205	0.88452	0.88289	0.55645
C206	0.61548	0.11712	0.94355
C207	0.58107	0.0959	0.944
N208	0.61892	0.1223	0.8315
C209	0.6343	0.12259	0.88479
C210	0.67406	0.1465	0.66291
C211	0.63697	0.1254	0.77382
C212	0.69637	0.17463	0.61059
C213	0.72654	0.21679	0.62569
C214	0.68639	0.16138	0.54937
H215	0.86006	0.90644	0.93141
H216	0.78461	0.91795	0.79016
H217	0.81726	0.93392	0.68978
H218	0.59366	0.1783	0.72412
H219	0.62746	0.20098	0.62534
H220	0.13994	0.59356	0.56859
H221	0.2226	0.71279	0.73144
H222	0.78185	0.20654	0.77552
H223	0.87254	0.20098	0.87466
H224	0.90634	0.1783	0.77588
H225	0.18274	0.43392	0.81022
H226	0.21539	0.41795	0.70984
H227	0.83681	0.87277	0.61485
H228	0.9331	0.91294	0.60149
H229	0.5669	0.08706	0.89851
H230	0.66319	0.12725	0.88515
C231	0.31361	0.33862	0.95063
C232	0.34239	0.38063	0.96814
C233	0.31153	0.39333	0.78604
C234	0.32964	0.40292	0.73103
C235	0.1215	0.65972	0.72175
C236	0.14003	0.67128	0.66698
C237	0.65762	0.11937	0.53186
C238	0.73522	0.22959	0.68576
C239	0.27346	0.71679	0.87431
C240	0.26478	0.72959	0.81424
C241	0.35997	0.67128	0.83302
C242	0.3785	0.65972	0.77825
C243	0.67036	0.90292	0.76897
C244	0.68847	0.89333	0.71396
C245	0.30363	0.32537	0.88941

C246	0.36303	0.3746	0.72618
C247	0.32594	0.3535	0.83709
C248	0.3657	0.37742	0.61521
N249	0.38108	0.37771	0.6685
C250	0.41893	0.4041	0.556
C251	0.38452	0.38289	0.55645
C252	0.11548	0.61712	0.94355
C253	0.08107	0.5959	0.944
N254	0.11892	0.6223	0.8315
C255	0.1343	0.62259	0.88479
C256	0.17406	0.6465	0.66291
C257	0.13697	0.6254	0.77382
C258	0.19637	0.67463	0.61059
C259	0.22654	0.71679	0.62569
C260	0.18639	0.66138	0.54937
H261	0.36006	0.40644	0.93141
H262	0.28461	0.41795	0.79016
H263	0.31726	0.43392	0.68978
H264	0.09366	0.6783	0.72412
H265	0.12746	0.70098	0.62534
H266	0.63994	0.09356	0.56859
H267	0.7226	0.21279	0.73144
H268	0.28185	0.70654	0.77552
H269	0.37254	0.70098	0.87466
H270	0.40634	0.6783	0.77588
H271	0.68274	0.93392	0.81022
H272	0.71539	0.91795	0.70984
H273	0.33681	0.37277	0.61485
H274	0.4331	0.41294	0.60149
H275	0.0669	0.58706	0.89851
H276	0.16319	0.62725	0.88515
C277	0.18639	0.16138	0.95063
C278	0.15761	0.11937	0.96814
C279	0.18847	0.10667	0.78604
C280	0.17036	0.09708	0.73103
C281	0.3785	0.84028	0.72175
C282	0.35997	0.82872	0.66698
C283	0.84238	0.38063	0.53186
C284	0.76478	0.27041	0.68576
C285	0.22654	0.78321	0.87431
C286	0.23522	0.77041	0.81424
C287	0.14003	0.82872	0.83302
C288	0.1215	0.84028	0.77825

C289	0.82964	0.59708	0.76897
C290	0.81153	0.60667	0.71396
C291	0.19637	0.17463	0.88941
C292	0.13697	0.1254	0.72618
C293	0.17406	0.1465	0.83709
C294	0.1343	0.12258	0.61521
N295	0.11892	0.12229	0.6685
C296	0.08107	0.0959	0.556
C297	0.11548	0.11711	0.55645
C298	0.38452	0.88288	0.94355
C299	0.41893	0.9041	0.944
N300	0.38108	0.8777	0.8315
C301	0.3657	0.87741	0.88479
C302	0.32594	0.8535	0.66291
C303	0.36303	0.8746	0.77382
C304	0.30363	0.82537	0.61059
C305	0.27346	0.78321	0.62569
C306	0.31361	0.83862	0.54937
H307	0.13994	0.09356	0.93141
H308	0.21539	0.08205	0.79016
H309	0.18274	0.06608	0.68978
H310	0.40634	0.8217	0.72412
H311	0.37254	0.79902	0.62534
H312	0.86006	0.40644	0.56859
H313	0.7774	0.28721	0.73144
H314	0.21815	0.79346	0.77552
H315	0.12746	0.79902	0.87466
H316	0.09366	0.8217	0.77588
H317	0.81726	0.56608	0.81022
H318	0.78461	0.58205	0.70984
H319	0.16319	0.12723	0.61485
H320	0.0669	0.08706	0.60149
H321	0.4331	0.91294	0.89851
H322	0.33681	0.87275	0.88515
C323	0.68639	0.66138	0.95063
C324	0.65761	0.61937	0.96814
C325	0.68847	0.60667	0.78604
C326	0.67036	0.59708	0.73103
C327	0.8785	0.34028	0.72175
C328	0.85997	0.32872	0.66698
C329	0.34238	0.88063	0.53186
C330	0.26478	0.77041	0.68576
C331	0.72654	0.28321	0.87431

C332	0.73522	0.27041	0.81424
C333	0.64003	0.32872	0.83302
C334	0.6215	0.34028	0.77825
C335	0.32964	0.09708	0.76897
C336	0.31153	0.10667	0.71396
C337	0.69637	0.67463	0.88941
C338	0.63697	0.6254	0.72618
C339	0.67406	0.6465	0.83709
C340	0.6343	0.62258	0.61521
N341	0.61892	0.62229	0.6685
C342	0.58107	0.5959	0.556
C343	0.61548	0.61711	0.55645
C344	0.88452	0.38288	0.94355
C345	0.91893	0.4041	0.944
N346	0.88108	0.3777	0.8315
C347	0.8657	0.37741	0.88479
C348	0.82594	0.3535	0.66291
C349	0.86303	0.3746	0.77382
C350	0.80363	0.32537	0.61059
C351	0.77346	0.28321	0.62569
C352	0.81361	0.33862	0.54937
H353	0.63994	0.59356	0.93141
H354	0.71539	0.58205	0.79016
H355	0.68274	0.56608	0.68978
H356	0.90634	0.3217	0.72412
H357	0.87254	0.29902	0.62534
H358	0.36006	0.90644	0.56859
H359	0.2774	0.78721	0.73144
H360	0.71815	0.29346	0.77552
H361	0.62746	0.29902	0.87466
H362	0.59366	0.3217	0.77588
H363	0.31726	0.06608	0.81022
H364	0.28461	0.08205	0.70984
H365	0.66319	0.62723	0.61485
H366	0.5669	0.58706	0.60149
H367	0.9331	0.41294	0.89851
H368	0.83681	0.37275	0.88515
N369	0.2959	0.18684	0.5
C370	0.36728	0.62653	0.5
C371	0.43623	0.58536	0.5
C372	0.4691	0.54252	0.5
H373	0.2754	0.21208	0.5
H374	0.48339	0.71025	0.48407

N375	0.7959	0.68684	0.5
C376	0.86728	0.12653	0.5
C377	0.93623	0.08536	0.5
C378	0.9691	0.04252	0.5
H379	0.7754	0.71208	0.5
H380	0.98229	0.21388	0.52294
N381	0.7041	0.81316	0.5
C382	0.63272	0.37347	0.5
C383	0.56377	0.41464	0.5
C384	0.5309	0.45748	0.5
H385	0.7246	0.78792	0.5
H386	0.51746	0.28975	0.48407
N387	0.2041	0.31316	0.5
C388	0.13272	0.87347	0.5
C389	0.06377	0.91464	0.5
C390	0.0309	0.95748	0.5
H391	0.2246	0.28792	0.5
H392	0.01641	0.78609	0.52666
N393	0.25	0.25	0.41308
H394	0.25	0.25	0.05443
N395	0.75	0.75	0.41308
H396	0.75	0.75	0.05443
N397	0.75	0.25	0.58692
H398	0.75	0.25	0.94557
N399	0.25	0.75	0.58692
H400	0.25	0.75	0.94557
N401	0.7959	0.18684	0
C402	0.36728	0.12655	0
C403	0.4691	0.04252	0
C404	0.43623	0.08535	0
H405	0.51611	0.79604	0.01482
N406	0.2959	0.68684	0
C407	0.86728	0.62655	0
C408	0.9691	0.54252	0
C409	0.93623	0.58535	0
H410	0.01628	0.29178	0.01486
N411	0.2041	0.81316	0
C412	0.63272	0.87345	0
C413	0.5309	0.95748	0
C414	0.56377	0.91465	0
H415	0.48333	0.20299	0.02224
N416	0.7041	0.31316	0
C417	0.13272	0.37345	0

C418	0.0309	0.45748	0
C419	0.06377	0.41465	0
H420	0.98361	0.70822	0.01486
N421	0.75	0.25	0.91308
N422	0.25	0.75	0.91308
N423	0.25	0.25	0.08692
N424	0.75	0.75	0.08692
C425	0.5	0.32522	0.5
C426	0.5	0.41511	0.5
C427	0	0.82522	0.5
C428	0	0.91511	0.5
C429	0.5	0.82522	0
C430	0.5	0.91511	0
C431	0	0.32522	0
C432	0	0.41511	0
H433	0.49891	0.71359	0.52704
H434	0.00091	0.21637	0.46747
H435	0.50057	0.79391	-0.02567
H436	0.00011	0.29475	-0.02973
H437	0.66037	0.35836	0.49833
H438	0.83963	0.14164	0.49833

Table S2. Fractional atomic coordinates for the unit cell of TUS-84 calculated based on the non-interpenetrated **scu** net.

Space group		*Pm*	
Calculated unit cell		*a* = 18.3743 Å, *b* = 50.3638 Å, *c* = 12.5042 Å, $\alpha = \beta = \gamma = 90°$	
atoms	x	y	z
C1	0.55004	0.2635	0.2658
C2	0.73967	0.3324	0.29249
C3	0.53782	0.82149	0.85637
C4	0.4814	0.84006	0.8519
C5	0.58171	0.33664	0.84708
C6	0.52639	0.35567	0.85026
C7	0.64462	0.29825	0.27255
C8	0.47537	0.35735	0.26474
C9	0.58753	0.31928	0.27167
C10	0.41199	0.39727	0.20926
N11	0.41325	0.37454	0.25828
C12	0.3428	0.4374	0.15233
C13	0.34306	0.4118	0.19626
C14	-0.06878	0.22856	0.76772
C15	-0.16459	0.19371	0.77803
H16	0.50173	0.27576	0.26516
H17	0.70564	0.34973	0.30325
H18	0.54129	0.80809	0.92368
H19	0.44176	0.84081	0.91584
H20	0.61943	0.33516	0.91335
H21	0.52195	0.36832	0.91975
H22	0.46125	0.40549	0.17498
H23	0.3937	0.447	0.13171
C24	0.55004	0.7635	0.7658
C25	0.73967	0.8324	0.79249
C26	0.53782	0.32149	0.35637
C27	0.4814	0.34006	0.3519
C28	0.58171	0.83664	0.34708
C29	0.52639	0.85567	0.35026
C30	0.64462	0.79825	0.77255
C31	0.47537	0.85735	0.76474
C32	0.58753	0.81928	0.77167
C33	0.41199	0.89727	0.70926
N34	0.41325	0.87454	0.75828

C35	0.3428	0.9374	0.65233
C36	0.34306	0.9118	0.69626
C37	-0.06878	0.72856	0.26772
C38	-0.16459	0.69371	0.27803
H39	0.50173	0.77576	0.76516
H40	0.70564	0.84973	0.80325
H41	0.54129	0.30809	0.42368
H42	0.44176	0.34081	0.41584
H43	0.61943	0.83516	0.41335
H44	0.52195	0.86832	0.41975
H45	0.46125	0.90549	0.67498
H46	0.3937	0.947	0.63171
C47	0.55004	0.7365	0.76558
C48	0.73967	0.6676	0.73889
C49	0.53782	0.17851	0.17501
C50	0.4814	0.15994	0.17948
C51	0.58171	0.66336	0.1843
C52	0.52639	0.64433	0.18112
C53	0.64462	0.70175	0.75883
C54	0.47537	0.64265	0.76664
C55	0.58753	0.68072	0.75971
C56	0.41199	0.60273	0.82212
N57	0.41325	0.62546	0.7731
C58	0.3428	0.5626	0.87905
C59	0.34306	0.5882	0.83512
C60	-0.06878	0.77144	0.26366
C61	-0.16459	0.80629	0.25335
H62	0.50173	0.72424	0.76622
H63	0.70564	0.65027	0.72813
H64	0.54129	0.19191	0.1077
H65	0.44176	0.15919	0.11554
H66	0.61943	0.66484	0.11803
H67	0.52195	0.63168	0.11163
H68	0.46125	0.59451	0.8564
H69	0.3937	0.553	0.89967
C70	0.55004	0.2365	0.26558
C71	0.73967	0.1676	0.23889
C72	0.53782	0.67851	0.67501
C73	0.4814	0.65994	0.67948
C74	0.58171	0.16336	0.6843
C75	0.52639	0.14433	0.68112
C76	0.64462	0.20175	0.25883
C77	0.47537	0.14265	0.26664

C78	0.58753	0.18072	0.25971
C79	0.41199	0.10273	0.32212
N80	0.41325	0.12546	0.2731
C81	0.3428	0.0626	0.37905
C82	0.34306	0.0882	0.33512
C83	-0.06878	0.27144	0.76366
C84	-0.16459	0.30629	0.75335
H85	0.50173	0.22424	0.26622
H86	0.70564	0.15027	0.22813
H87	0.54129	0.69191	0.6077
H88	0.44176	0.65919	0.61554
H89	0.61943	0.16484	0.61803
H90	0.52195	0.13168	0.61163
H91	0.46125	0.09451	0.3564
H92	0.3937	0.053	0.39967
C93	0.00342	0.7365	0.2658
C94	-0.18621	0.6676	0.29249
C95	0.01564	0.17851	0.85637
C96	0.07206	0.15994	0.8519
C97	-0.02825	0.66336	0.84708
C98	0.02706	0.64433	0.85026
C99	-0.09116	0.70175	0.27255
C100	0.07809	0.64265	0.26474
C101	-0.03407	0.68072	0.27167
C102	0.14146	0.60273	0.20926
N103	0.14021	0.62546	0.25828
C104	0.21065	0.5626	0.15233
C105	0.2104	0.5882	0.19626
C106	0.62223	0.77144	0.76772
C107	0.71805	0.80629	0.77803
H108	0.05173	0.72424	0.26516
H109	-0.15218	0.65027	0.30325
H110	0.01217	0.19191	0.92368
H111	0.1117	0.15919	0.91584
H112	-0.06597	0.66484	0.91335
H113	0.03151	0.63168	0.91975
H114	0.09221	0.59451	0.17498
H115	0.15975	0.553	0.13171
C116	0.00342	0.2365	0.7658
C117	-0.18621	0.1676	0.79249
C118	0.01564	0.67851	0.35637
C119	0.07206	0.65994	0.3519
C120	-0.02825	0.16336	0.34708

C121	0.02706	0.14433	0.35026
C122	-0.09116	0.20175	0.77255
C123	0.07809	0.14265	0.76474
C124	-0.03407	0.18072	0.77167
C125	0.14146	0.10273	0.70926
N126	0.14021	0.12546	0.75828
C127	0.21065	0.0626	0.65233
C128	0.2104	0.0882	0.69626
C129	0.62223	0.27144	0.26772
C130	0.71805	0.30629	0.27803
H131	0.05173	0.22424	0.76516
H132	-0.15218	0.15027	0.80325
H133	0.01217	0.69191	0.42368
H134	0.1117	0.65919	0.41584
H135	-0.06597	0.16484	0.41335
H136	0.03151	0.13168	0.41975
H137	0.09221	0.09451	0.67498
H138	0.15975	0.053	0.63171
C139	0.00342	0.2635	0.76558
C140	-0.18621	0.3324	0.73889
C141	0.01564	0.82149	0.17501
C142	0.07206	0.84006	0.17948
C143	-0.02825	0.33664	0.1843
C144	0.02706	0.35567	0.18112
C145	-0.09116	0.29825	0.75883
C146	0.07809	0.35735	0.76664
C147	-0.03407	0.31928	0.75971
C148	0.14146	0.39727	0.82212
N149	0.14021	0.37454	0.7731
C150	0.21065	0.4374	0.87905
C151	0.2104	0.4118	0.83512
C152	0.62223	0.22856	0.26366
C153	0.71805	0.19371	0.25335
H154	0.05173	0.27576	0.76622
H155	-0.15218	0.34973	0.72813
H156	0.01217	0.80809	0.1077
H157	0.1117	0.84081	0.11554
H158	-0.06597	0.33516	0.11803
H159	0.03151	0.36832	0.11163
H160	0.09221	0.40549	0.8564
H161	0.15975	0.447	0.89967
C162	0.00342	0.7635	0.26558
C163	-0.18621	0.8324	0.23889

C164	0.01564	0.32149	0.67501
C165	0.07206	0.34006	0.67948
C166	-0.02825	0.83664	0.6843
C167	0.02706	0.85567	0.68112
C168	-0.09116	0.79825	0.25883
C169	0.07809	0.85735	0.26664
C170	-0.03407	0.81928	0.25971
C171	0.14146	0.89727	0.32212
N172	0.14021	0.87454	0.2731
C173	0.21065	0.9374	0.37905
C174	0.2104	0.9118	0.33512
C175	0.62223	0.72856	0.76366
C176	0.71805	0.69371	0.75335
H177	0.05173	0.77576	0.26622
H178	-0.15218	0.84973	0.22813
H179	0.01217	0.30809	0.6077
H180	0.1117	0.34081	0.61554
H181	-0.06597	0.83516	0.61803
H182	0.03151	0.86832	0.61163
H183	0.09221	0.90549	0.3564
H184	0.15975	0.947	0.39967
N185	-0.22327	0.20993	0.77016
N186	-0.22327	0.70993	0.27016
N187	-0.22327	0.79007	0.26122
N188	-0.22327	0.29007	0.76122
N189	-0.11352	0.25	0.76569
N190	-0.11352	0.75	0.26569
N191	0.66698	0.75	0.76569
N192	0.66698	0.25	0.26569
C193	0.27673	0.60079	0.81336
C194	0.27673	0.52419	0.95919
C195	0.27673	0.54976	0.90094
C196	0.27673	0.10079	0.31336
C197	0.27673	0.02419	0.45919
C198	0.27673	0.04976	0.40094
C199	0.27673	0.39921	0.21802
C200	0.27673	0.47581	0.07219
C201	0.27673	0.45024	0.13044
C202	0.27673	0.89921	0.71802
C203	0.27673	0.97581	0.57219
C204	0.27673	0.95024	0.63044
H205	0.71363	0.75	0.26569
H206	0.71363	0.25	0.76569

H207	-0.16017	0.25	0.26569
H208	-0.16017	0.75	0.76569
H209	0.24621	0.47954	0.7879
H210	0.27673	0.61973	0.78163
H211	0.2347	0.97924	0.2869
H212	0.27673	0.11973	0.28163
H213	0.24059	0.52004	0.28551
H214	0.27673	0.38027	0.24975
H215	0.24175	0.02065	0.7721
H216	0.27673	0.88027	0.74975
C217	0.27673	0.5	0.78284
C218	0.27673	0.5	0.9028
C219	0.27673	0.5	0.24854
C220	0.27673	0.5	0.12858
H221	0.32547	0.5	0.69049
H222	0.33039	0.5	0.29261
C223	0.27673	0	0.28284
C224	0.27673	0	0.4028
C225	0.27673	0	0.74854
C226	0.27673	0	0.62858
H227	0.31876	0	0.19476
H228	0.32919	0	0.80275

Table S3. Fractional atomic coordinates for the unit cell of TUS-84 calculated based on the **csq** net.

Space group		*P6/mmm*	
Calculated unit cell		*a* = *b* = 52.3706 Å, *c* = 18.6277 Å, *α* =*β* = 90°, *γ* = 120°	
atoms	x	y	z
C1	0.27226	0.50297	0.35226
C2	0.27088	0.51551	0.28786
C3	0.29429	0.496	0.37042
C4	0.31725	0.47965	0.46311
C5	0.2993	0.49074	0.44194
C6	0.34353	0.5119	0.313
C7	0.36047	0.51099	0.25598
C8	0.34666	0.49256	0.19657
C9	0.31591	0.4751	0.19541
C10	0.29905	0.47563	0.25277
C11	0.31266	0.49426	0.31175
C12	0.39016	0.4987	0.13374
N13	0.36284	0.49246	0.13454
C14	0.43136	0.49966	0.06512
C15	0.4042	0.49874	0.0654
H16	0.28687	0.52332	0.24473
H17	0.32907	0.47231	0.42909
H18	0.35443	0.52674	0.35749
H19	0.38413	0.52539	0.25743
H20	0.305	0.46107	0.14967
H21	0.27528	0.46186	0.25071
H22	0.40261	0.50318	0.18312
C23	0.50009	0.55514	1
C24	0.50187	0.60929	1
C25	0.5	0.52693	1
C26	0.52689	0.52689	1
C27	0.55557	0.55557	1
N28	0.50245	0.71155	0.5
N29	0.50434	0.75217	0.39155
H30	0.51723	0.75861	0.43778

13. Liquid NMR spectra of building blocks

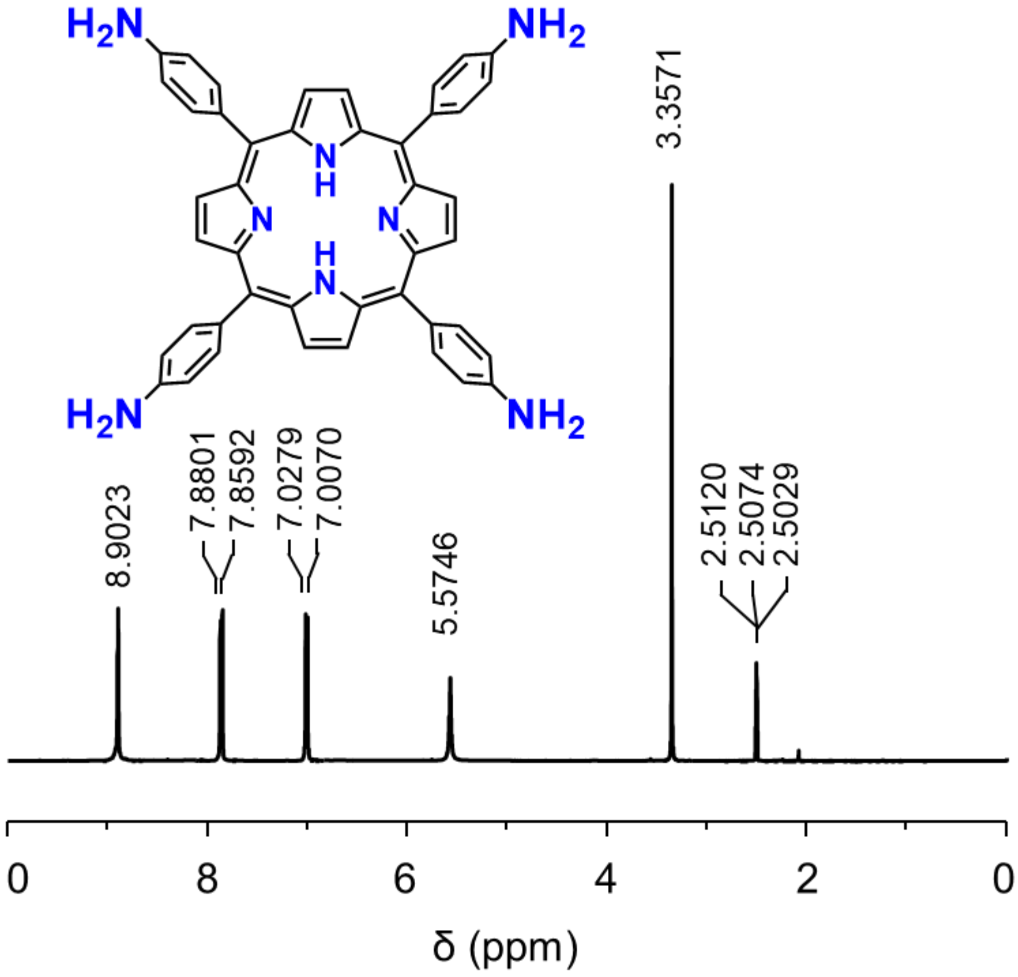

Figure S30: ^{1}H NMR spectrum of TAPP in DMSO-d_6.

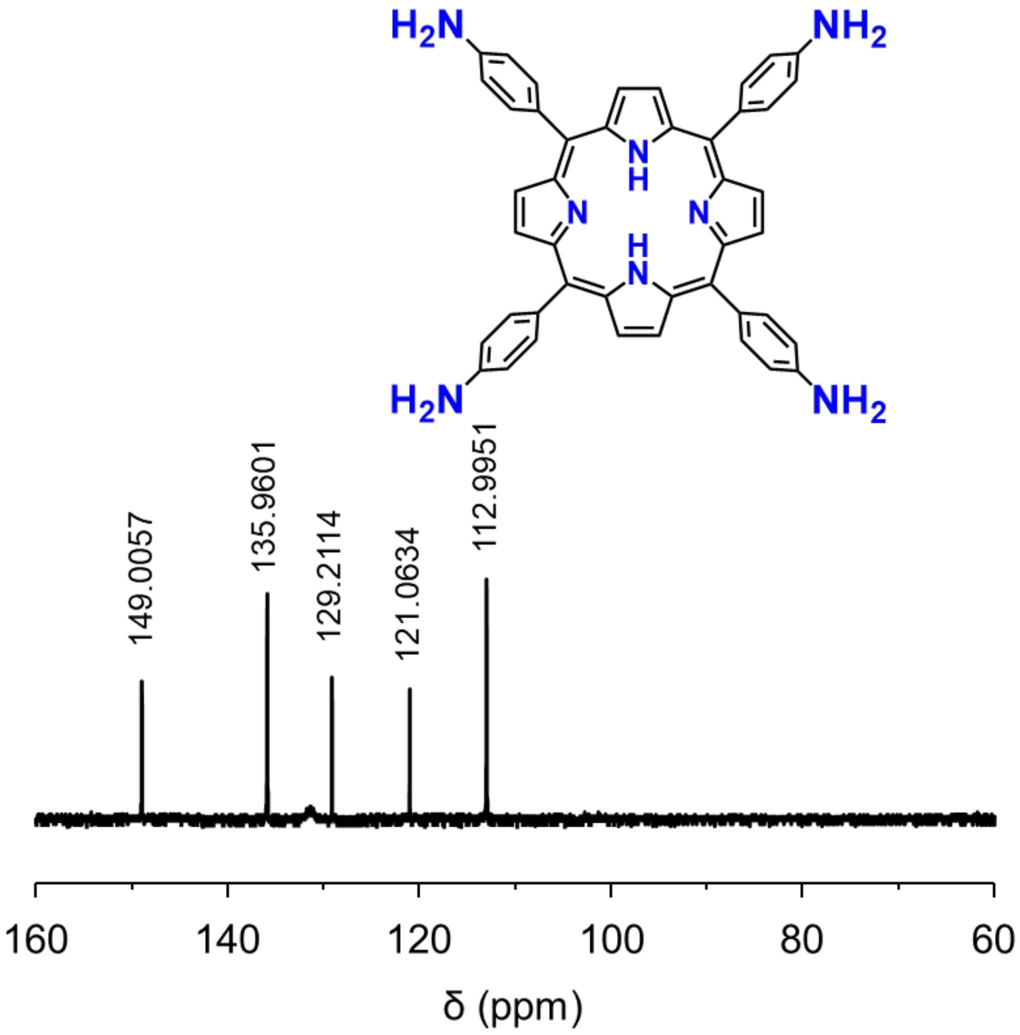

Figure S31: ^{13}C NMR spectrum of TAPP in DMSO-d$_6$.

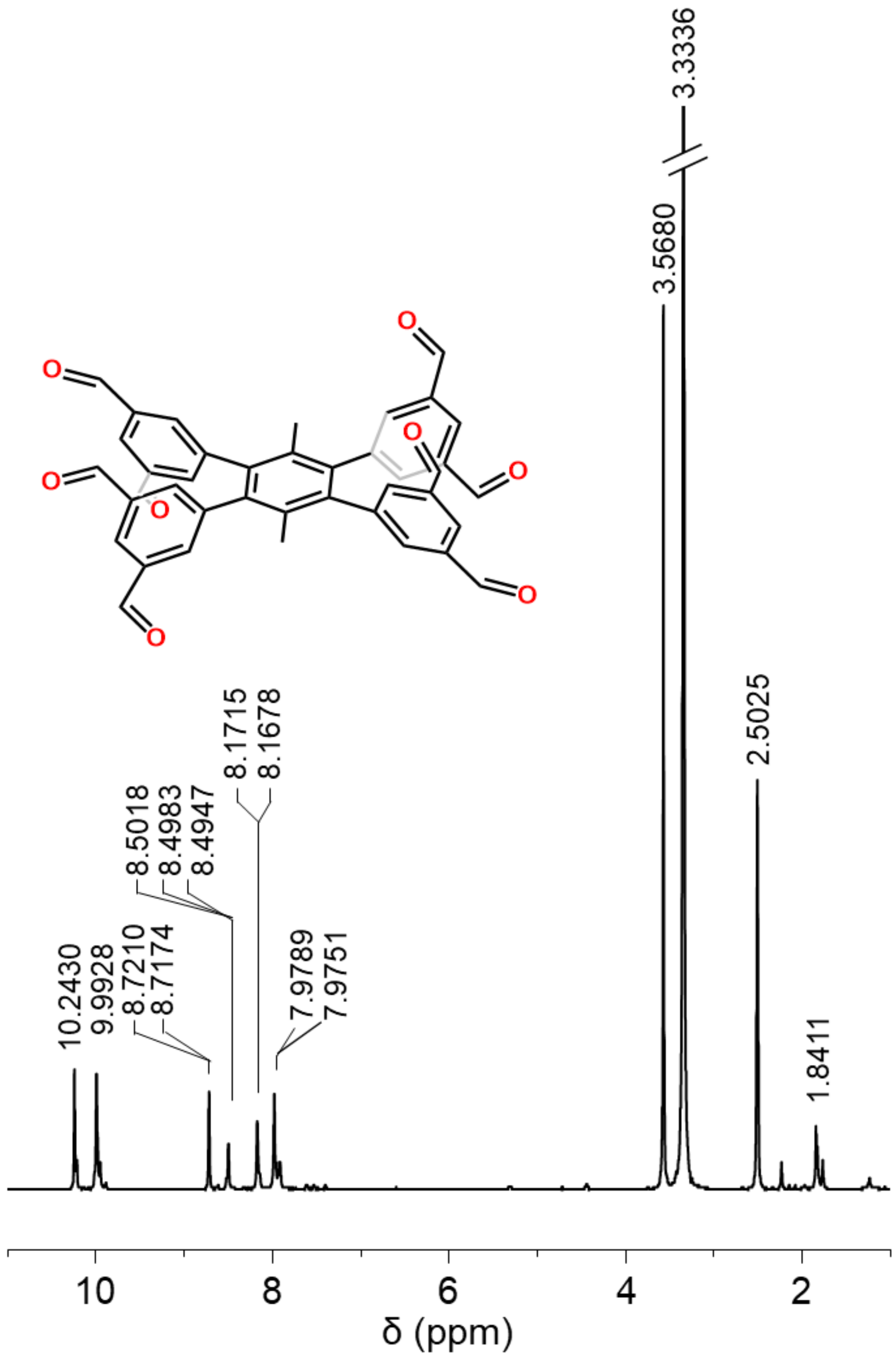

Figure S32: ^{1}H NMR spectrum of DPTB-Me in DMSO-d_6.

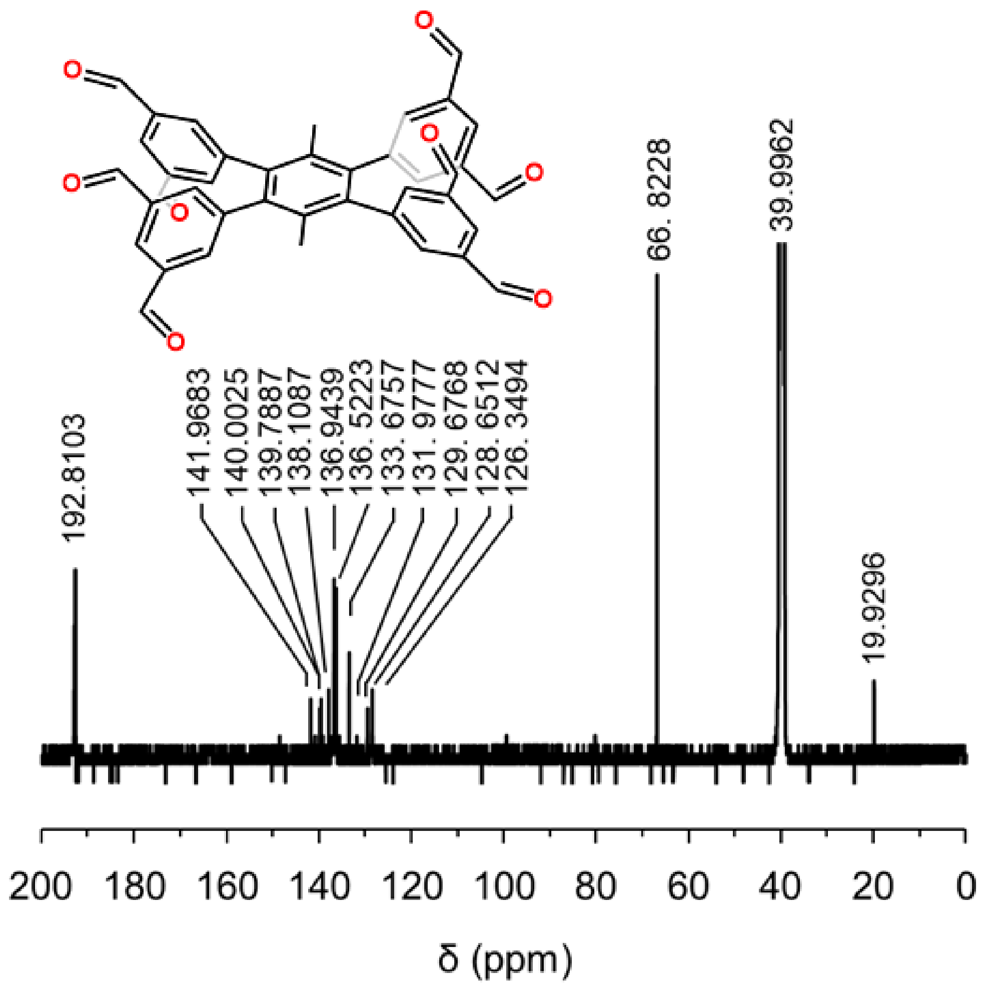

Figure S33: ^{13}C NMR spectrum of DPTB-Me in DMSO-d_6.

14. References

(1) Jin, F.; Lin, E.; Wang, T.; Geng, S.; Wang, T.; Liu, W.; Xiong, F.; Wang, Z.; Chen, Y.; Cheng, P.; Zhang, Z. Bottom-Up Synthesis of 8-Connected Three-Dimensional Covalent Organic Frameworks for Highly Efficient Ethylene/Ethane Separation. *J. Am. Chem. Soc.* **2022**, *144*, 5643-5652.

(2) Yuasa, M.; Oyaizu, K.; Yamaguchi, A.; Kuwakado, M. Micellar Cobaltporphyrin Nanorods in Alcohols. *J. Am. Chem. Soc.* **2004**, *126*, 11128-11129.

(3) Bettelheim, A.; White, B. A.; Raybuck, S. A.; Murray, R. W. Electrochemical polymerization of amino-, pyrrole-, and hydroxy-substituted tetraphenylporphyrins. *Inorg. Chem.* **1987**, *26*, 1009-1017.

(4) Das, S.; Ben, T.; Qiu, S.; Valtchev, V. Two-Dimensional COF−Three-Dimensional MOF Dual-Layer Membranes with Unprecedentedly High H_2/CO_2 Selectivity and Ultrahigh Gas Permeabilities. *ACS Appl. Mater. Interfaces* **2020**, *12*, 52899-52907.
